# Model-based evaluation of the impact of prophylactic vaccination applied to Ebola epidemics in Sierra Leone and Democratic Republic of Congo

**DOI:** 10.1186/s12879-022-07723-6

**Published:** 2022-10-04

**Authors:** Ravi Potluri, Amit Kumar, Valérie Oriol-mathieu, Thierry Van Effelterre, Laurent Metz, Hitesh Bhandari

**Affiliations:** 1 SmartAnalyst Inc., 300 Vesey Street, 10th Floor, New York, NY 10282 USA; 2 SmartAnalyst India Pvt. Ltd., Gurugram, India; 3 Janssen Vaccines & Prevention B.V., Leiden, The Netherlands; 4 Janssen Pharmaceutica N.V., Beerse, Belgium; 5 Johnson & Johnson Global Public Health, New Brunswick, NJ USA

**Keywords:** Ebola, Impact, Model, Transmission, Vaccination

## Abstract

**Background:**

Protection by preventive Ebola vaccines has been demonstrated in clinical trials, but a complete picture of real-world effectiveness is lacking. Our previous study modeling the impact of preventively vaccinating healthcare workers (HCW) alone or with a proportion of the general population (GP) estimated significant reductions in incidence and mortality. The model assumed 100% vaccine efficacy, which is unlikely in the real world. We enhanced this model to account for lower vaccine efficacy and to factor in reduced infectiousness and lower case fatality rate in vaccinated individuals with breakthrough infections.

**Methods:**

The previous model was enhanced to still permit a risk, although lower, for vaccinated individuals to become infected. The enhanced model, calibrated with data from epidemics in Sierra Leone (SL) and North Kivu, Democratic Republic of the Congo, helped evaluate the impact of preventive Ebola vaccination in different scenarios based on different vaccine efficacy rates (90% and 30% reductions in infection risk in the base and conservative scenarios, respectively; additionally, both scenarios with 50% reductions in infectiousness and mortality) and vaccination coverage among HCWs (30%, 90%) and GP (0%, 5%, and 10%).

**Results:**

The base scenario estimated that, depending upon the proportions of vaccinated HCWs and GP, 33–85% of cases and 34–87% of deaths during the 2014 SL epidemic and 42–89% of cases and 41–89% of deaths during the 2018 North Kivu epidemic would be averted versus no vaccination. Corresponding estimates for the conservative scenario were: 23–74% of cases and 23–77% of deaths averted during the SL epidemic and 31–80% of both cases and deaths averted during the North Kivu epidemic.

**Conclusions:**

Preventive vaccination targeting HCW alone or with GP may significantly reduce the size and mortality of an EVD outbreak, even with modest efficacy and coverage. Vaccines may also confer additional benefits through reduced infectiousness and mortality in breakthrough cases.

**Supplementary Information:**

The online version contains supplementary material available at 10.1186/s12879-022-07723-6.

## Background

Ebola virus disease (EVD), a severe and often fatal infectious disease, is caused by the zoonotic Ebolavirus, a member of the family filoviridae, with *Zaire ebolavirus* as the most common species [1, 2]. Ebola virus (EBOV) has caused at least 30 outbreaks and epidemics in African regions close to the equator since first being reported in 1976, with the Democratic Republic of the Congo (DRC) alone having faced 12 outbreaks thus far [3, 4]. The largest outbreak to date occurred between 2013 and 2016 and started in Guinea, from where it spread to other African countries including Liberia, Mali, Senegal, and Sierra Leone (SL), with cases spreading to the United States and Europe, eventually resulting in a total of 28,652 cases and 11,317 deaths [2]. Ebola remains a constant and unpredictable threat to a number of sub-Saharan Africa countries.

The clinical presentation is often severe with systemic spread and multiorgan dysfunction [1, 2]. Case fatality rates (CFRs) are variable but typically greater than 50% [2]. Survivors of EVD frequently suffer long-term sequelae with nonspecific manifestations. Some of these manifestations are, (a) fatigue and weight gain, (b) mental health issues such as anxiety disorders and depression, and (c) impacts on multiple organ systems resulting in a range of cardiac, visual, aural, skeletal, and muscular abnormalities [2, 5]. It is also suspected that the virus persists for a long duration in immunologically privileged reservoirs (eye, central nervous system, and semen), exposing the patients to EVD relapse, and the population to new outbreaks [4].

Outbreaks of EVD often begin with introduction of the virus into humans from an unknown reservoir through an unknown route, following which transmission between humans occurs by direct contact or through contact with infected tissues, bodily fluids, or contaminated fomites [6–8]. But phylogenetic analyses of viruses in the two most recent outbreaks in Guinea and DRC have shown that new outbreaks can likely spread from survivors still carrying dormant virus [9]. Healthcare workers, as also the other frontline workers, at the forefront of EVD patient care constitute the main group at increased risk of infection with EBOV and death from EVD during outbreaks [10, 11]. The overall impact of EVD extends well beyond the significant morbidity and high mortality in those directly affected by the disease. The disease has an indirect impact on population health given the need to divert valuable and frequently scarce resources from other health programs including those intended to treat human immunodeficiency virus (HIV) infections, tuberculosis, and African trypanosomiasis and those intended to ensure maternal and infant health and provide primary care [5]. Further, outbreaks of EVD have a significant social impact, as seen during the West Africa epidemic of 2013–2016, which resulted in food insecurity, closure of educational institutions, and loss of one or both parents of young children [12]. This epidemic also had a global economic impact in the form of loss of gross domestic product (GDP) between $2.8 and $32.6 billion and imposing an estimated overall economic and social burden of $53.19 billion (including $18.8 billion due to deaths from non-EVD causes) [12].

Several treatment options including convalescent plasma, anti-EBOV glycoprotein monoclonal antibody cocktails, and antiviral agents (remdesivir [GS-5734] and favipravir) are currently being evaluated in clinical trials. Two monoclonal antibodies (Inmazeb and Ebanga) were approved for the treatment of *Zaire ebolavirus* (Ebolavirus) infection in adults and children by the US Food and Drug Administration in late 2020. With or without other treatments, intensive supportive care in the form of fluid/electrolyte replacement and medication to support blood pressure, reduce vomiting and diarrhea, and to manage fever/pain can significantly improve chances of survival when provided early [1, 2, 13, 14]. Despite the progress made in managing EVD outbreak, the approaches still face various obstacles. Such obstacles highlighted the need of effective preventive interventions such as vaccination.

Several EBOV vaccine candidates are currently in different stages of development, with a few having received approvals or marketing authorizations [1, 2]. While some of these are only licensed in the country of origin (e.g., China [Ad5-EBOV]; Russia [EpivacEbola, GamEvacCombi, and GamEvacLyo]), others have recently been approved or granted marketing authorizations by the United States Food and Drug Administration (FDA) (rVSVΔG-ZEBOV-GP–ERVEBO®), the European Medicines Agency (EMA) (rVSVΔG-ZEBOV-GP and Ad26.ZEBOV–ZABDENO®, MVA-BN-Filo–MVABEA® vaccine regimen) and been pre-qualified by the WHO (rVSVΔG-ZEBOV-GP and Ad26.ZEBOV, MVA-BN-Filo vaccine regimen). The rVSVΔG-ZEBOV-GP is a single-dose vaccine based on a live, attenuated recombinant vesicular stomatitis virus (VSV) vector platform [1, 2]. It is indicated for protection against the *Zaire ebolavirus* species of EBOV [15, 16]. The Ad26.ZEBOV, MVA-BN-Filo vaccine regimen is a two-component vaccine designed to induce long-term immunity. The initial dose of Ad26.ZEBOV, based on a single recombinant, replication-incompetent human adenovirus type 26 vectored vaccine, is followed by a dose of MVA-BN-Filo, a recombinant, non-replicating in human cells, Modified Vaccinia Ankara-Bavarian Nordic (MVA-BN) vectored multivalent Filovirus vaccine [1, 2]. This two-dose regimen received marketing authorization under exceptional circumstances from the European commission in July 2020 [17] and is indicated for the prevention of EVD caused by the *Zaire ebolavirus* species in individuals ≥ 1 year-old.

The Strategic Advisory Group of Experts (SAGE) on Immunization of the World Health Organization (WHO) used the findings of impact modeling, available empirical data, and field-level knowledge to recommend in 2019 the adoption of a ring vaccination based on contact tracing and vaccination of at-risk individuals, namely healthcare workers (HCW) including frontline workers (FLW), with rVSVΔG-ZEBOV-GP complemented by vaccination with the Ad26.ZEBOV, MVA-BN-Filo vaccine regimen in populations at lower risk [18]. Recently this strategy was confirmed in case of outbreak by way of vaccination of individuals (e.g., HCWs and FLWs) with Ad26.ZEBOV, MVA-BN-Filo vaccination in neighboring areas and countries where the outbreak may spread [19]. Implementation of a ring vaccination strategy in the midst of an outbreak can be complicated by multiple factors including security issues, nosocomial transmission, weak healthcare infrastructure, and a highly mobile target population [18]. Furthermore and for the first time, the updated SAGE recommendation has proposed a pre-emptive vaccination with Ad26.ZEBOV, MVA-BN-Filo vaccine regimen, in the absence of any outbreak, for national response teams, international responders, Ebola laboratory workers and specialized Ebola research & treatment unit workers while not immediately recommending a broader population use [19]. The current study focuses on assessing the impact of such preemptive vaccination among high-risk groups such as HCW.

Recurring outbreaks, challenges faced during outbreaks, and risk of spread to nearby geographic areas underscore the urgent need to proactively vaccinate high-risk individuals in areas in and around the outbreak zones with a vaccine designed to induce long-term immunity. With the aim of preventing the spread of the 2019 outbreak beyond disease-stricken areas within the DRC through its border with the Republic of Rwanda, several thousands of doses of Ad26.ZEBOV and MVA-BN-Filo were committed for use in individuals at risk of contracting EVD. Similarly, a large deployment study is being implemented in West-Africa where, in order to limit the spread of the outbreak in Guinea beyond borders, the Ministry of Health and Sanitation in Sierra Leone is leading the exercise to administer the Ad26.ZEBOV, MVA-BN-Filo vaccine regimen on compassionate grounds with technical, logistical, and operational support from the WHO and the U.S Centers for Disease Control and Prevention (CDC) [20, 21].

While the durability of immune responses elicited by the Ad26.ZEBOV, MVA-BN-Filo vaccine regimen against EBOV has been demonstrated in clinical trials [22–24], its effectiveness is yet to be demonstrated with real-world data. Epidemiological modeling has been previously recognized and recommended by the SAGE of the WHO as a tool to assess the potential impacts of various vaccination strategies [25]. In an attempt to facilitate decision making by key stakeholders on the need for a prophylactic vaccine, we previously modeled the impact of prophylactic vaccination of varying proportions of both HCW (doctors, nurses, and midwives) and the general non-HCW population (the susceptible population other than the HCW) on the size of a potential EVD outbreak by simulating the 2014 epidemic in SL [26]. Our study modeled the ability of vaccination to reduce the risk of infection under the assumption that the vaccine would be 100% effective. In the real world, vaccine effectiveness may not reach 100%, resulting in some vaccinated individuals to be at risk of being infected. However, studies of other vaccines [27, 28] as well as recent studies of patients with EVD [29, 30] have shown that individuals who get infected despite being vaccinated have lower viral loads than unvaccinated individuals and that they have reduced infectiousness, lower disease severity, and better clinical outcomes.

Given this context, the current study was carried out to study the impact of vaccination if vaccine efficacy is less than 100%. At the same time, we enhanced the previously constructed model [26] to capture the effects of reduced infectiousness and lower CFRs that results in a less severe form of EVD in vaccinated individuals that contract the infection. These evaluations were carried out in two models calibrated to real historic outbreaks: the SL epidemic of 2014 and the DRC (North Kivu) epidemic of 2018.

## Methods

### Model structure

**Fig. 1:**
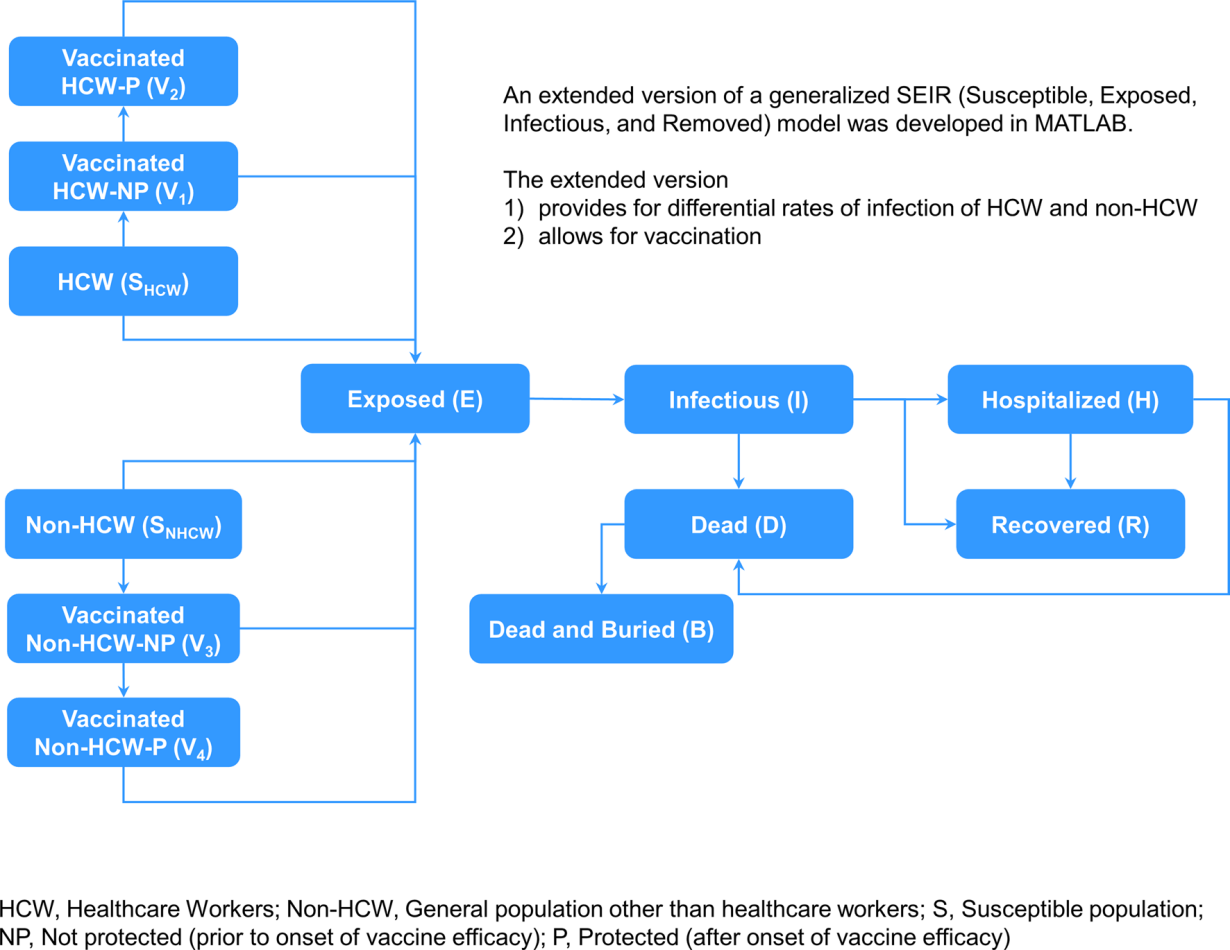
SEIR framework-based model differentiating between HCW and the general non-HCW population, permitting transitions between populations based on infection status, and accommodating vaccination

The model used in our previous study [26] was based on a standard generalized ‘susceptible, exposed, infected, and removed (SEIR)’ model, enhanced to facilitate differentiation between HCW and the general population and to allow transitions between susceptible, exposed, and infected populations while also accounting for preventive and reactive vaccination (Fig. 1). Following calibration to a prior EVD outbreak (the 2014 epidemic in SL), the adapted model permitted assessment of the benefits of preventive vaccination in different scenarios where HCW and members of the general population are vaccinated at differential coverages, while assuming the vaccine to be 100% effective in reducing the risk of being infected.

### Model enhancement

**Fig. 2:**
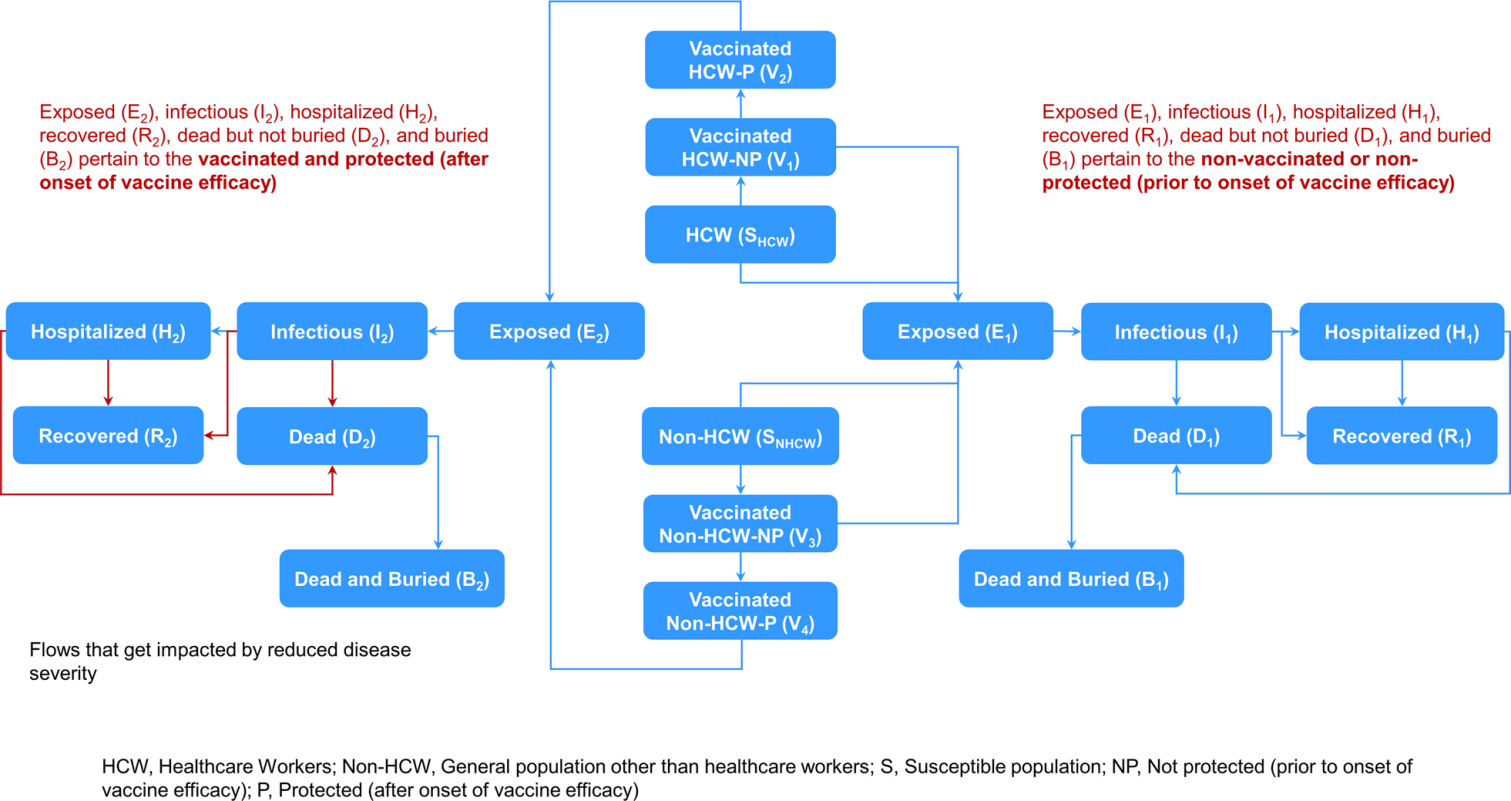
Enhanced model structure to capture reduction in infectiousness and case fatality rate of disease as an added benefit of vaccination

The current study accounts for a vaccine efficacy that may be less than 100%. The existing model was therefore enhanced to allow vaccinated individuals to still have a risk, although lower, of becoming infected. Additionally, the model was further improved to factor in the benefits of vaccination that confer a reduced risk of infectiousness and a lower CFR of EVD in previously vaccinated infected individuals. This was done by replicating each of the post-exposure compartments (exposed, E; infectious but not hospitalized, I; infectious and hospitalized, H; recovered, R; dead but not buried, D; and buried, B) (Fig. 2), with one set comprising previously unvaccinated individuals or those vaccinated but who became exposed prior to the onset of vaccine efficacy (denoted with the suffix “1”) and the other set comprising previously vaccinated individuals who were exposed after the onset of vaccine efficacy (denoted with the suffix “2”). The model structure and transitions for the enhanced model are detailed in Additional file 1: Supplementary material A1.

### Model calibration

The current study involved assessment of the impact of vaccination in two different modeled outbreaks.

For the outbreak modeled on the 2014 EVD epidemic in SL, the details of model calibration have been previously discussed by Potluri et al. [26], with the model parameters derived being presented in Table 1.

**Table 1:** Model fitting using individual and disease/epidemic-specific parameters

Parameter	Description	2014 Sierra Leone Epidemic				2018 DRC (North Kivu) Epidemic					
		Days 0–95	Days 95–186	Days 186–587	Source	Days 0–120	Days 120–217	Days 217–273	Days 273–385	Days 385–546	Source
N	Size of the total population at time 0	7,017,144 Sierra Leone (2014)			World Bank Group [52]	8,300,000 North Kivu province of DRC (2018)					UN-OCHA [53]
S_HCW_	HCW at time 0	1153			Evans, 2015 [11]	5230					Barroy, 2014 [51]
1/σ	Mean latency period	7 days	7 days	7 days	Legrand, 2007 [54]	7 days	7 days	7 days	7 days	7 days	Meakin, 2018 [55]
1/γ_D_	Mean duration from death to burial	2 days	2 days	2 days	Legrand, 2007 [54]	2 days	2 days	2 days	2 days	2 days	
1/α	Mean duration from onset of infection to hospitalization	2.4 days	2.4 days	2.2 days	Fitted	5.0 days	5.0 days	4.0 days	3.5 days	3.5 days	Fitted
β_I→HCW_	Transmission rate from infectious individuals to HCW (In 1/days)	117.8	15.0	5.1	Fitted	30.0	13.0	7.0	6.5	3.8	Fitted
β_H→HCW_	Transmission rate from hospitalized individuals to HCW (In 1/days)	189.21	23.64	8.88	Fitted	37.0	16.0	10.0	8.0	4.2	Fitted
β_D→HCW_	Transmission rate from dead but not buried individuals to HCW (In 1/days)	0.0726	0.0511	0.0450	Fitted	0.035	0.032	0.030	0.030	0.030	Fitted
β_I→NHCW_	Transmission rate from infectious individuals to the non-HCW/general population (In 1/days)	0.635	0.594	0.425	Fitted	0.2700	0.2500	0.4000	0.2780	0.2557	Fitted
β_H→NHCW_	Transmission rate from hospitalized individuals to the non-HCW/general population (In 1/days)	0.0020	0.0010	0.0005	Fitted	0.0160	0.0140	0.0200	0.0165	0.0160	Fitted
β_D→NHCW_	Transmission rate from dead but not buried individuals to the non-HCW/general population (In 1/days)	0.0726	0.0511	0.0450	Fitted	0.035	0.032	0.030	0.030	0.030	Fitted
δ_1_	Case fatality rate among non-hospitalized infectious individuals	0.46	0.21	0.68	Fitted	0.67	0.67	0.80	0.68	0.50	Fitted
δ_2_	Case fatality rate among hospitalized individuals	0.46	0.21	0.68	Fitted	0.67	0.67	0.80	0.68	0.50	Fitted
1/γ	Mean duration from onset of infection to death/recovery	6 days	6 days	6 days	Fitted	9.6 days	9.6 days	9.6 days	9.6 days	9.6 days	Meakin, 2018 [55]
1/γ_H_	Mean duration from hospitalization to death/recovery	6.2 days	8.3 days	16 days	Fitted	4.6 days	4.6 days	7.0 days	8.0 days	8.2 days	Fitted
K_1_	Proportion of HCW in the total population at the start of the epidemic	0.016%			Calculated (Evans, 2015) [11]	0.063%					Calculated (Barroy, 2014) [51]

DRC: Democratic Republic of the Congo; HCW: healthcare workers

**Fig. 3:**
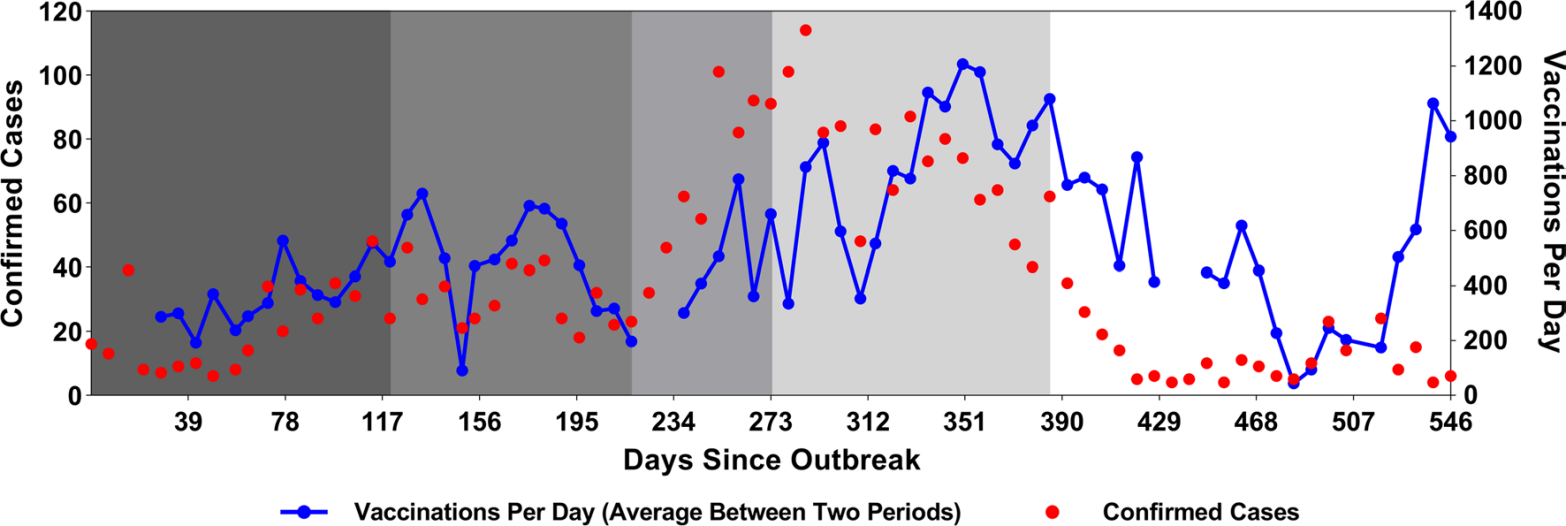
New Ebola cases in North Kivu plotted against the rate of vaccination (with the rVSVΔG-ZEBOV-GP Ebola vaccine) in DRC as reported by WHO

For the outbreak modeled on the 2018 epidemic in the North Kivu province of the DRC, model calibration was performed according to data obtained between August 5, 2018 and February 2, 2020, divided into five periods (Table 1): (i) Day 0 to Day 119, in which an initial spurt of EVD cases in North Kivu showed an incremental trend in weekly cases, during which a ring vaccination program was initiated on August 8, 2018 [31]; (ii) Days 120 to 217, during which 30 to 50 cases of EVD were reported weekly during a downturn in the vaccination drive, as indicated by the reduction in daily vaccination rate; (iii) Days 218 to 273, when an increasing trend in weekly EVD cases was witnessed amidst a revival of the vaccination drive, as demonstrated by an increase in the rate of vaccination; (iv) Days 274 to 385, when weekly cases began to decline as the vaccination rate reached its peak (> 1200 vaccinations daily); and (v) Days 386 to 546, a period characterized by a sharp decline in weekly cases (Fig. 3).

The model parameters derived from the 2014 SL epidemic and the 2018 DRC (North Kivu) epidemic were then used for stochastic simulation of respective outbreaks by applying the direct method algorithm described by Gillespie [32]. The cumulative cases and deaths from these two models (the model calibrated with data from the 2014 SL epidemic and the one calibrated with data from the North Kivu epidemic) were compared with WHO data relating to the respective outbreaks to check for closeness of fit.

### Model basic reproduction number

For the enhanced model calibrated using data from the 2014 SL epidemic, there were no changes in the parameters required for estimation of basic reproduction number (R_0_), and it therefore remained unchanged from the original analysis.

For the enhanced model calibrated using the 2018 North Kivu epidemic in the DRC, the approach from the original study based on data from the 2014 SL epidemic was used where the next generation matrix described by Diekmann et al. in 1990 [33] was applied to calculate R_0_ according to the following equation:

$${R}_{0} = \left[\frac{{K}_{1}{\beta }_{I\to HCW}+\left(1-{K}_{1}\right){\beta }_{I\to NHCW}}{\left(\alpha \right.+\left.\gamma \right)}+\alpha \left(\frac{{K}_{1}{\beta }_{H\to HCW}+\left(1-{K}_{1}\right){\beta }_{H\to NHCW}}{{\gamma }_{H}\left(\alpha \right.+\left.\gamma \right)}\right)+\frac{{\beta }_{D}\left({\delta }_{1}\gamma + \left.{\delta }_{2}\alpha \right)\right.}{\left(\alpha \right.+\left.\gamma \right){\gamma }_{D}}\right]$$

K_1_, Proportion of HCW in the total population at the start of the epidemic; β_*I*→*HCW*_, Transmission rate from infectious individuals to HCW (In 1/days); β_*I*→NH*CW*_, Transmission rate from infectious individuals to NHCW (In 1/days); $1/\mathrm{\alpha }$, Mean duration from onset of infection to hospitalization; 1/γ, Mean duration from onset of infection to death/recovery; β_H→H*CW*,_ Transmission rate from hospital isolated infectious individuals to HCW (In 1/days); β_H→NH*CW*,_ Transmission rate from hospital isolated infectious individuals to NHCW (In 1/days); 1/γ_H_, Mean duration from hospitalization to death/recovery; 1/γ_D,_ Mean duration from death to burial; δ_1_, Case fatality rate among non-hospitalized infectious individuals; δ_2_, Case fatality rate among hospitalized individual.

A more detailed derivation of R_0_ can be found in Additional file 1: Supplementary material A1.

### Scenarios analyzed

The enhanced model described above, calibrated with data from the 2014 SL epidemic and the 2018 North Kivu epidemic in the DRC, was used to conduct evaluations of the impact of vaccination based on different vaccine efficacy scenarios and levels of vaccine coverage among HCW and the general population.

The vaccine efficacy scenarios included: (i) a base scenario with a 90% reduction in the risk of infection; and (ii) a conservative scenario in which the risk of infection was reduced by 30%. In both scenarios, the infectiousness and case fatality rate were reduced by 50%.

As for levels of vaccine coverage, a systematic review of vaccination coverage of HCW in Africa against Hepatitis B reported a range of vaccination coverage between 13.4% (central Africa) and 62.1% (northern Africa) [34]. Given this large variability, we considered a wide range of vaccination coverage rates: 30% and 90% among HCW and 0%, 5%, and 10% among the general non-HCW population.

A total of 8 scenarios based on different combinations of the above vaccine efficacy and coverage inputs were evaluated for each of the two models (Table 2). In all of these scenarios, it was assumed that vaccinated individuals received prophylactic vaccination before the beginning of the outbreak and that vaccine efficacy did not decrease over the course of the outbreak.

**Table 2:** Scenarios based on different combinations of vaccine efficacy, HCW vaccination coverage and GP vaccination coverage

Scenario	Vaccine efficacy		HCW coverage (%)	General population coverage (%)	
	Reduced risk of being infected (%)	Reduced infectiousness and case fatality rate (%)			
1	90	50	30%	0	Base scenarios for vaccine efficacy
2	90	50	90%	0	
3	90	50	30	5	
4	90	50	30	10	
5	30	50	30	0	Conservative scenarios for vaccine efficacy
6	30	50	90	0	
7	30	50	30	5	
8	30	50	30	10	

GP, general population; HCW, healthcare workers

## Results

### Model calibration

**Fig. 4:**
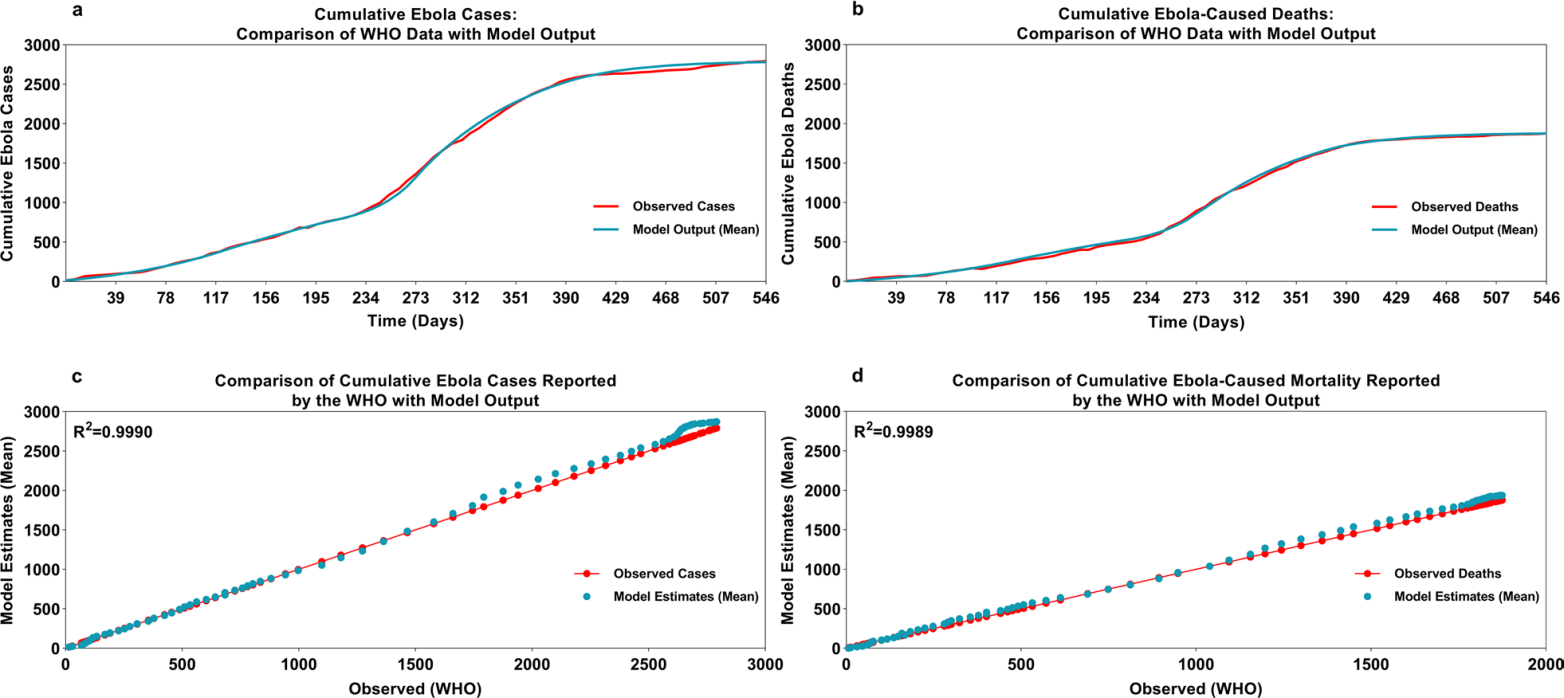
Cumulative Ebola cases and deaths in North Kivu, model output versus observed cases by WHO

Results of the calibration exercise for the model calibrated using data from the 2014 SL epidemic have been previously reported by Potluri et al. [26]. For the model calibrated using data from the 2018 North Kivu epidemic, comparison of the output of the stochastic simulation of the fitted model with data reported by the WHO confirmed a good fit (Fig. 4A–D). The model simulated 2782 cases and 1876 deaths, compared with 2791 cumulative confirmed cases and 1875 deaths reported by the WHO in its situation report of February 4, 2020 (data reported as of February 2, 2020) [35].

In the original exercise using data from the 2014 SL epidemic [26], R_0_ was calculated to be 1.34. This is consistent with previously published estimates of R_0_ for the 2014 SL epidemic (1.26 < R_0_ < 2.53) [36]. Similarly, R_0_ for the scenario without any intervention or vaccination using the model based on data from the 2018 epidemic in North Kivu in the DRC was calculated to be 1.08.

### Impact of prophylactic vaccination: base scenario for vaccine efficacy

**Fig. 5:**
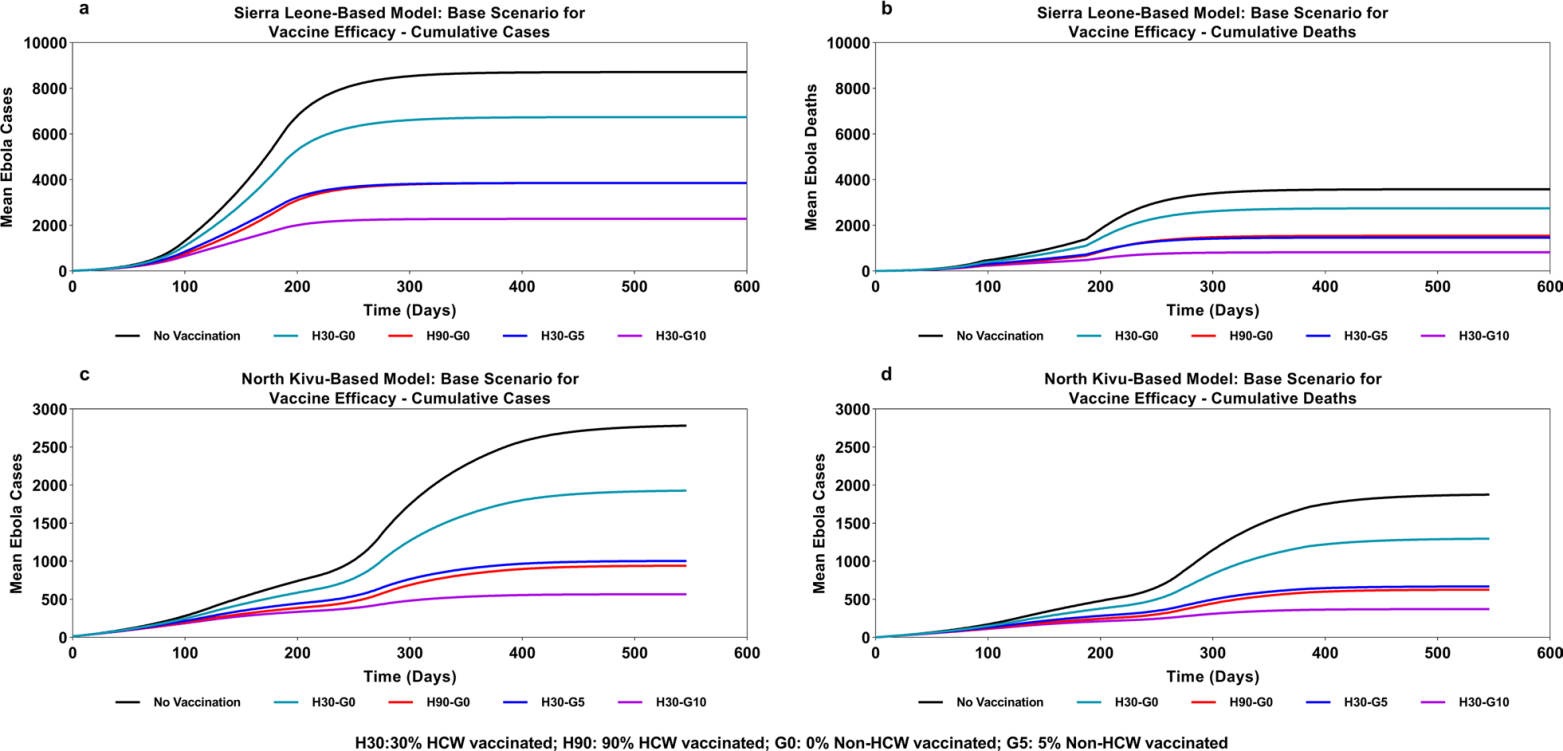
Impact of prophylactic vaccination of healthcare workers and general population on cumulative incidence and mortality associated with Ebola Virus Disease—base scenario for vaccine efficacy

When comparing an epidemic in the “no vaccination” scenario with one in which prophylactic vaccination (base scenario of vaccine efficacy of 90% reduced risk of infection and 50% reduced infectiousness and case fatality rate) of some HCW was carried out, the calibrated model estimated that prophylactic vaccination of 346 HCW in SL (representing 30% of all HCW and just 0.005% of the overall population) would help avert EVD cases by 33% (2892 cases averted) and deaths by 34% (1204 deaths averted). Corresponding vaccination of 30% HCW in North Kivu in the DRC (1569 HCW, representing 0.02% of the overall population) was estimated to help avert the number of cases by 42% (1158 cases averted) and deaths by 41% (778 deaths averted). By assuming much greater prophylactic vaccination coverage—of 90% of all HCW, the corresponding reduction was projected to be 71% (6175 cases and 2559 deaths averted) in the SL-based model and 79% (2206 cases and 1486 deaths averted) in the DRC (North Kivu)-based model (Fig. 5 and Table 3).

**Table 3:** Impact of prophylactic vaccination of HCW and GP on burden of EVD-base scenario

Parameter	2014 Sierra Leone Epidemic					2018 DRC (North Kivu) Epidemic				
	No vaccination	30% of HCW vaccinated	90% of HCW vaccinated	30% of HCW + 5% of GP vaccinated	30% of HCW + 10% of GP vaccinated	No vaccination	30% of HCW vaccinated	90% of HCW vaccinated	30% of HCW + 5% of GP vaccinated	30% of HCW + 10% of GP vaccinated
Number vaccinated	0	346	1038	351,145	701,944	0	1569	4707	416,307	831,044
Cumulative cases (IQR; 95%CI)	8711 (4420–11,946; 597–22,026)	5819 (2680–8100; 207–15,985)	2536 (919–3627; 51–7838)	2594 (1114–3623; 87–7432)	1271 (499–1797; 50–3699)	2782 (700–4026; 54–9637)	1624 (296–2410; 46–6072)	576 (115–762; 36–2620)	645 (148–901; 40–2630)	315 (104–416; 36–1180)
Proportion of cases averted vs no vaccination (IQR; 95%CI)	–	33% (32–40%; 26–62%)	71% (69–79%; 63–93%)	70% (70–75%; 66–84%)	85% (85–89%; 82–93%)	–	42% (39–52%; 15–59%)	79% (76–86%; 33–87%)	77% (75–81%; 26–82%)	89% (85–90%; 33–90%)
Proportion of cases averted vs vaccination of 30% of HCW (IQR; 95%CI)	–	–	–	55% (55–58%; 52–63%)	78% (78–81%; 74–85%)	–	–	–	60% (50–63%; 14–65%)	81% (65–82%; 22–83%)
Cumulative deaths (IQR; 95%CI)	3580 (1834–4901; 246–9000)	2376 (1081–3305; 75–6576)	1021 (362–1465; 22–3189)	985 (418–1384; 37–2816)	463 (182–657; 22–1343)	1876 (477–2722; 36–6453)	1098 (198–1627; 30–4097)	390 (76–515; 24–1785)	439 (99–614; 26–1796)	213 (70–281; 24–806)
Proportion of deaths averted vs no vaccination (IQR; 95%CI)	–	34% (33–41%; 27–62%)	71% (70–80%; 64–92%)	72% (72–77%; 68–84%)	87% (87–90%; 84–92%)	–	41% (39–52%; 17–59%)	79% (76–86%; 34–87%)	77% (74–81%; 29–82%)	89% (85–90%; 34–90%)
Proportion of deaths averted vs vaccination of 30% of HCW (IQR; 95%CI)	–	–	–	59% (58–61%; 51–64%)	81% (80–82%; 71–85%)	–	–	–	60% (50–63%; 14–65%)	81% (65–82%; 21–83%)

Base scenario for vaccine efficacy: 90% reduced risk of being infected and 50% reduced infectiousness and case fatality rate

CI: credible interval; DRC: Democratic Republic of the Congo; EVD: ebola virus disease; GP: general population; HCW: healthcare workers; IQR: inter-quartile range

When part of the general population was also vaccinated prophylactically along with the HCW, the numbers of cases and deaths averted were substantially greater. Vaccination of 10% of the general population, in addition to the vaccination of 30% of all HCW, was predicted to curtail the number of cases by 85% (7440 cases averted) and mortality by 87% (3117 deaths averted) versus the “no vaccination” scenario in the SL-based model, and by 89% cases (2467 cases averted) and 89% deaths (1663 deaths averted) versus the “no vaccination” scenario in the DRC (North Kivu)-based model. If instead only 5% of the general population were vaccinated, to add to the vaccination of 30% of HCW, the model predicted the reduction in the numbers of cases and deaths by 70% and 72%, respectively, in the SL-based model, and both cases and deaths by 77% in the DRC (North Kivu)-based model (Fig. 5 and Table 3).

### Impact of prophylactic vaccination: conservative scenario for vaccine efficacy

**Fig. 6:**
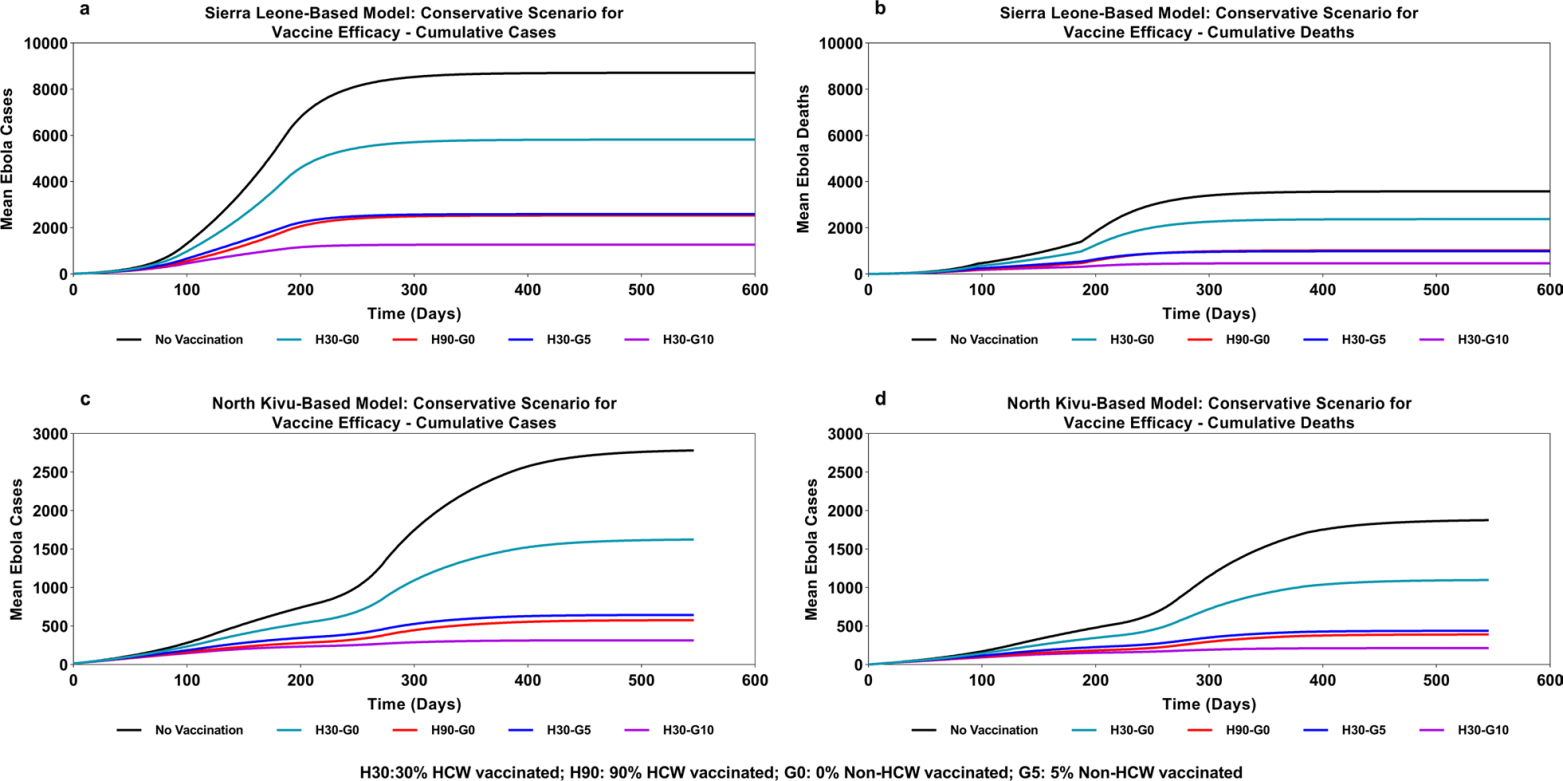
Impact of prophylactic vaccination of healthcare workers and general population on cumulative incidence and mortality associated with Ebola Virus Disease—conservative scenario for vaccine efficacy

Even with a conservative assumption of vaccine efficacy (30% reduced risk of infection and 50% reduced infectiousness and case fatality rate), prophylactic vaccination of 30% of HCW was estimated to help reduce both cases and deaths in the overall population by 23% in the SL-based model (as compared with 33% cases and 34% deaths in the base case), and by 31% in the DRC (North Kivu)-based model (as compared with 42% cases and 41% deaths in the base case). Assuming prophylactic vaccination of 90% of all HCW, the corresponding reduction was projected to be 56% in the number of cases and 59% in the number of deaths in the SL-based model, with the DRC (North Kivu)-based model estimating a 64% reduction in numbers of both cases and deaths (Fig. 6 and Table 4).

**Table 4:** Impact of prophylactic vaccination of HCW and GP on burden of EVD-conservative scenario

Parameter	2014 Sierra Leone Epidemic					2018 DRC (North Kivu) Epidemic				
	No vaccination	30% of HCW vaccinated	90% of HCW vaccinated	30% of HCW + 5% of GP vaccinated	30% of HCW + 10% of GP vaccinated	No vaccination	30% of HCW vaccinated	90% of HCW vaccinated	30% of HCW + 5% of GP vaccinated	30% of HCW + 10% of GP vaccinated
Number vaccinated	0	346	1038	351,145	701,944	0	1569	4707	416,307	831,044
Cumulative cases (IQR; 95%CI)	8711 (4420–11,946; 597–22,026)	6740 (3242–9413; 240–17,667)	3856 (1578–5432; 77–11,239)	3855 (1774–5426; 136–10,635)	2285 (978–3183; 70–6589)	2782 (700–4026; 54–9637)	1928 (344–2862; 49–7295)	942 (167–1326; 43–4061)	1004 (204–1448; 47–3848)	567 (147–799; 41–2105)
Proportion of cases averted vs no vaccination (IQR; 95%CI)	–	23% (21–27%; 19–55%)	56% (54–64%; 47–84%)	56% (55–60%; 51–74%)	74% (73–78%; 69–87%)	–	31% (28–43%; 12–51%)	66% (63–76%; 20–79%)	64% (62–70%; 13–73%)	80% (78–82%; 24–83%)
Proportion of cases averted vs vaccination of 30% of HCW (IQR; 95%CI)	–	–	–	43% (42–45%; 36–46%)	66% (66–70%; 61–75%)	–	–	–	48% (40–51%; 5–53%)	71% (57–72%; 14–73%)
Cumulative deaths (IQR; 95%CI)	3580 (1834–4901; 246–9000)	2749 (1316–3865; 89–7192)	1544 (626–2177; 32–4545)	1466 (668–2064; 54–4049)	821 (350–1152; 29–2391)	1876 (477–2722; 36–6453)	1297 (231–1922; 31–4877)	627 (107–885; 28–2700)	669 (133–968; 30–2578)	371 (94–524; 26–1384)
Proportion of deaths averted vs no vaccination (IQR; 95%CI)	–	23% (22–28%; 19–57%)	57% (56–65%; 48–84%)	59% (58–63%; 54–75%)	77% (76–81%; 73–88%)	–	31% (28–43%; 14–51%)	67% (64–76%; 24–80%)	64% (62–71%; 18–74%)	80% (78–83%; 29–84%)
Proportion of deaths averted vs vaccination of 30% of HCW (IQR; 95%CI)	–	–	–	47% (46–49%; 38–50%)	70% (70–73%; 65–77%)	–	–	–	48% (42–51%; 6–54%)	71% (59–73%; 17–74%)

Conservative scenario for vaccine efficacy: 30% reduced risk of being infected and 50% reduced infectiousness and case fatality rate

CI: credible interval; DRC: Democratic Republic of the Congo; EVD: ebola virus disease; GP: general population; HCW: healthcare workers; IQR: inter-quartile range

The impact was more pronounced when even a small proportion of the general population was also vaccinated prophylactically along with the HCW. Vaccination of 10% of the general population, in addition to vaccination of 30% of all HCW, was predicted to limit the epidemic to 2285 cases and 821 deaths (74% reduction in cases and 77% reduction in deaths versus the “no vaccination” scenario) in the SL-based model and 567 cases and 371 deaths (80% reduction versus the “no vaccination” scenario) in the DRC (North Kivu)-based model. Moreover, vaccinating only 5% of the general population along with 30% of HCW was predicted to limit the epidemic by reducing the numbers of cases and deaths versus the “no vaccination” scenario by 56% and 59%, respectively, in the SL-based model and by 64% each in the DRC (North Kivu)-based model (Fig. 6 and Table 4).

### Impact of reduced infectiousness and case fatality rate for the most conservative scenario

**Fig. 7:**
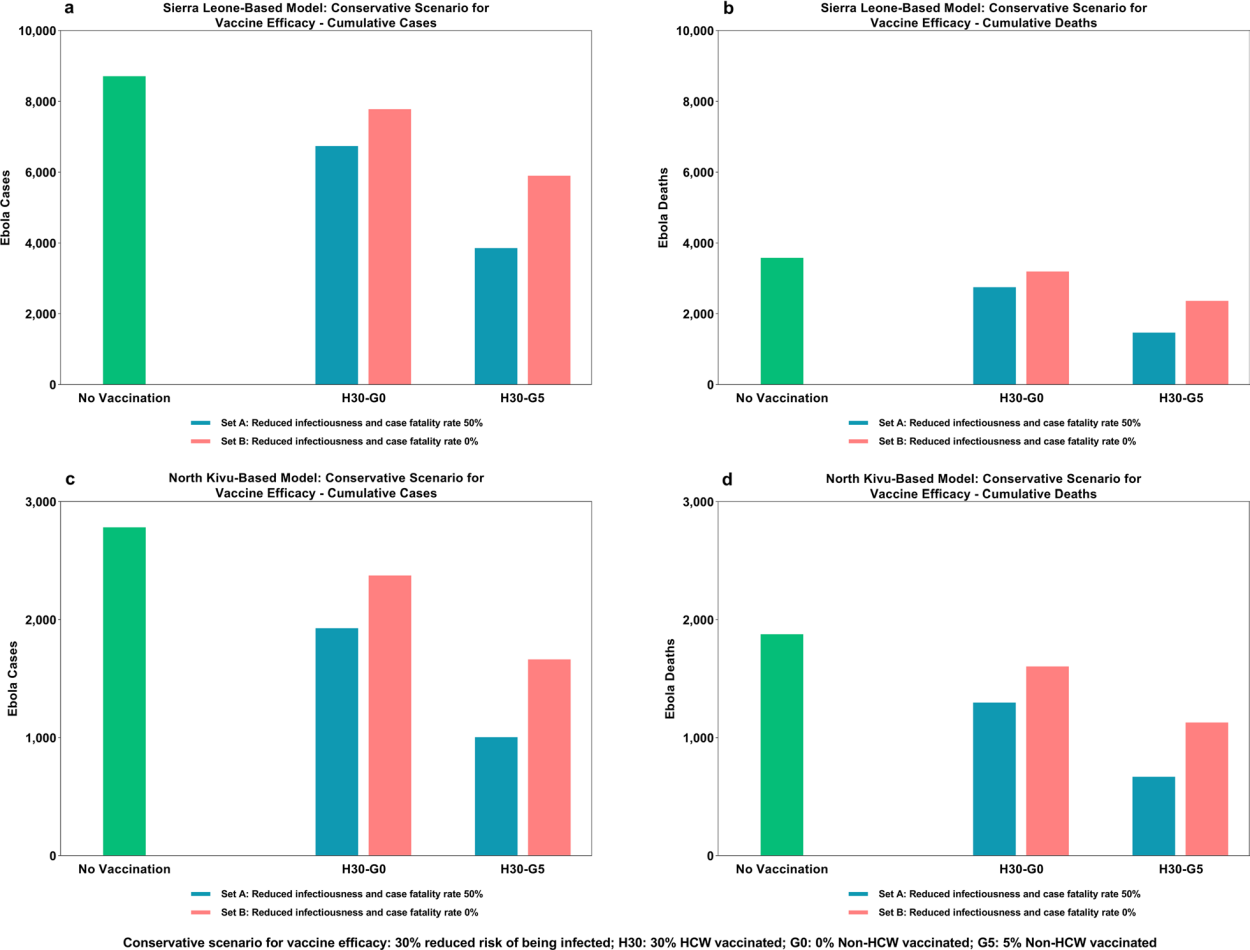
Impact of additional vaccine efficacy in the form of 50% reduction in infectiousness and case fatality rate

To this conservative scenario in which vaccination resulted in both a reduced risk of infection (30% VE) and a reduction in infectiousness and case fatality rate (50%; Set A in Fig. 7), we compared a scenario in which vaccination resulted in a reduced risk of infection (30% VE) but not in a reduction in infectiousness or case fatality rate (Set B in Fig. 7), in order to isolate the impact of reduced infectiousness and case fatality rate in vaccinated but infected cases. Both scenarios were individually compared with a ‘no vaccination’ scenario. There were fewer cases and deaths in the Set A model. We determined that with a vaccine that reduces the risk of infection and mortality as well as infectiousness (Set A), vaccination of 30% of HCW (and no members of the general population) would help reduce both cases and deaths by 23% in the SL-based model (compared with an 11% reduction in both cases and deaths in Set B) and by 31% in the DRC (North Kivu)-based model (compared with a 15% reduction in both cases and deaths in Set B). Similarly, vaccination of 5% of the general population in addition to 30% of HCW resulted in a reduction of 56% of cases (vs 32%) and 59% of deaths (vs 34%) in the SL-based model, and 64% of cases and deaths (vs 40% of cases and deaths) in the DRC (North Kivu)-based model.

## Discussion

The largest epidemic of EVD in recorded history, which occurred in Western Africa between 2013 and 2016, claimed more than 11,300 lives out of 28,652 cases [2]. The second worst outbreak of EVD to date, which occurred in the DRC between 2018 and 2020, claimed nearly 2300 lives out of 3481 cases for a mortality rate of 66% [37], reminding the global community of the urgent need for proactive preventive measures. It has been seen in recent EVD outbreaks that the disease may emerge because of increased contact between humans and wildlife, with extensive deforestation, hunting, and mining being factors contributing to this increased contact [3] or from EVD survivors who still carry the virus in some reservoirs even years later, potentially leading to new EVD cases transmitted through exposure of infected body fluids of EVD survivors [38, 39]. However, given the lack of adequate understanding of the introduction of EBOV into humans from animal reservoir [2], the small number of documented cases of re-introduction from survivors [9], and the unpredictability of this event, it is difficult to anticipate when and where the next outbreak may occur. Moreover, the risk of having more frequent outbreaks is a concern with increasing social mobility and urbanization, making preventive immunization one of the best strategies to defend against this unpredictable pathogen [40].

The accelerated development and approval of vaccines [15, 17, 22, 24, 41–44], especially during the West Africa EVD epidemic of 2013–2016, provide the tools for the prevention of EVD [1, 2, 45]. The Global Health Security Agenda—a multilateral global initiative—includes immunization as one of the 11 key areas of focus [46]. Optimizing the healthcare systems of countries at risk for an EVD outbreak is clearly a priority [10]. This can be achieved through infection prevention measures among HCWs through enhancement of occupational safety in healthcare settings and by ensuring prophylactic vaccination of HCWs and other relevant populations.

In the current modelling study, prophylactic vaccination was evaluated based on different vaccine efficacy scenarios and varying levels of vaccination coverage of HCW and the general population. All scenarios, in both the SL- and DRC (North Kivu)-based models, showed substantial benefits of prophylactic vaccination of HCW as measured by the reduction in number of cases and deaths. As we further demonstrated, reducing the infectiousness and case fatality rate by 50%, in a conservative vaccine efficacy scenario (reduction in risk of being infected) of only 30% while prophylactically vaccinating 30% of HCW and 5% of the general population, would result in reducing the number of cases in the SL-based model by more than half and by nearly two-thirds in the DRC (North Kivu)-based model. A more effective vaccine (90% reduced risk of being infected), would have an even higher likelihood of containing an EVD outbreak (70% reduction in cases in the SL-based model and 77% reduction in the DRC [North Kivu]-based model).

While broad vaccination of a population could potentially avoid Ebola outbreaks, we considered an alternate strategy in line with the current SAGE recommendation, in which high-risk populations such as HCW would be targeted on a priority basis. Even in a conservative vaccine efficacy scenario (30% reduced risk of being infected and 50% reduced infectiousness and case fatality rate), vaccinating 90% of all HCW (without vaccination in the general population) would result in substantial reduction in cases of EVD (56% in the SL-based model and 66% in the DRC [North Kivu]-based model) and deaths (57% in the SL-based model and 67% in the DRC [North Kivu]-based model). Given the high risk of infection that HCW carry, coupled with the ease of coverage of this small-sized population, prophylactically vaccinating this population would result in substantial benefits in terms of cases averted per vaccination. Across all evaluations with base and conservative scenarios, modeling prophylactic vaccination of only HCW would result in approximately 4700 to 8400 fewer cases per 1000 vaccinations in the SL-based model and approximately 400 to 700 fewer cases per 1000 vaccinations in the DRC (North Kivu)-based model (Table 5). These findings are aligned with previously published models that analyzed the impact of vaccinating HCWs [36, 47–50]. While we included only physicians, nurses, and midwives in our analysis of prophylactic vaccination among high-risk groups, inclusion of a wider group of other frontline workers such as drivers, security personnel, and other non-medical staff at high risk of exposure to EVD could further enhance the impact of a prophylactic vaccination strategy. Further extending prophylactic vaccination to also include part of the general population shows great promise. Prophylactic vaccination of just 5% of the general population in the conservative case vaccine efficacy scenarios coupled with vaccination of 30% of HCW is projected to more than double the benefit in preventing the numbers of cases (4856 vs 1971 in SL-based model and 1778 vs 854 in DRC (North Kivu)-based model) and deaths (2114 vs 831 in SL-based model and 1207 vs 579 in DRC (North Kivu)-based model).

**Table 5:** Cases averted per 1000 vaccinations vs ‘no vaccination’

Vaccine efficacy	Sierra Leone 2014				DRC 2018 (North Kivu)			
	30%		90%		30%		90%	
Non-HCW	HCW							
	H30 (346)	H90 (1038)	H30 (346)	H90 (1038)	H30 (1569)	H90 (4707)	H30 (1569)	H90 (4707)
G0 (0)	5,698	4,679	8,361	5,951	544	391	738	469
G5 (351K)	13.8		17.4		4		5	
G10 (702K)	9.2		10.6		3		3	

No vaccination: Cumulative cases = 8711 in Sierra Leone 2014 and 2782 in DRC 2018 (North Kivu), Cumulative deaths = 3580 in Sierra Leone 2014 and 1876 in DRC 2018 (North Kivu)

(Number of vaccinations in parentheses); DRC: Democratic Republic of the Congo; HCW: healthcare workers; H30: 30% HCW vaccinated; H90: 90% HCW vaccinated; G0: 0% Non-HCW vaccinated; G5: 5% Non-HCW vaccinated; G10: 10% Non-HCW vaccinated

### Comparison of vaccines with and without additional efficacy of 50% reduction in infectiousness and case fatality rate

Our study demonstrated greater impact on cases and deaths with a vaccine that offers reduction in infectiousness and case fatality rate among individuals who are infected despite getting vaccinated. We compared model scenarios where vaccination helped reduce the risk of being infected as well as reduced infectiousness and case fatality rates (Set A) with another set of scenarios where vaccination only reduced the risk of infection but did not confer any reduction in infectiousness and case fatality rates (Set B). The reduction in number of cases was greater for Set A scenarios compared with Set B scenarios (23% in Set A compared to 11% in SL-based model and 31% vs 15% in DRC (North Kivu)-based model in a scenario involving 30% vaccination of HCW and no vaccination of the general population). This is attributed to the additional beneficial effect of vaccination at the population level due to reduced infectiousness of infected individuals. We expect that the incremental impact will be consistent across vaccination scenarios, regardless of whether only high-risk HCW are vaccinated, or if the general population is also vaccinated along with HCW (Fig. 7).

### Comparison of Ebola model based on the 2014 epidemic in SL and the 2018 epidemic in DRC (North Kivu)

**Fig. 8:**
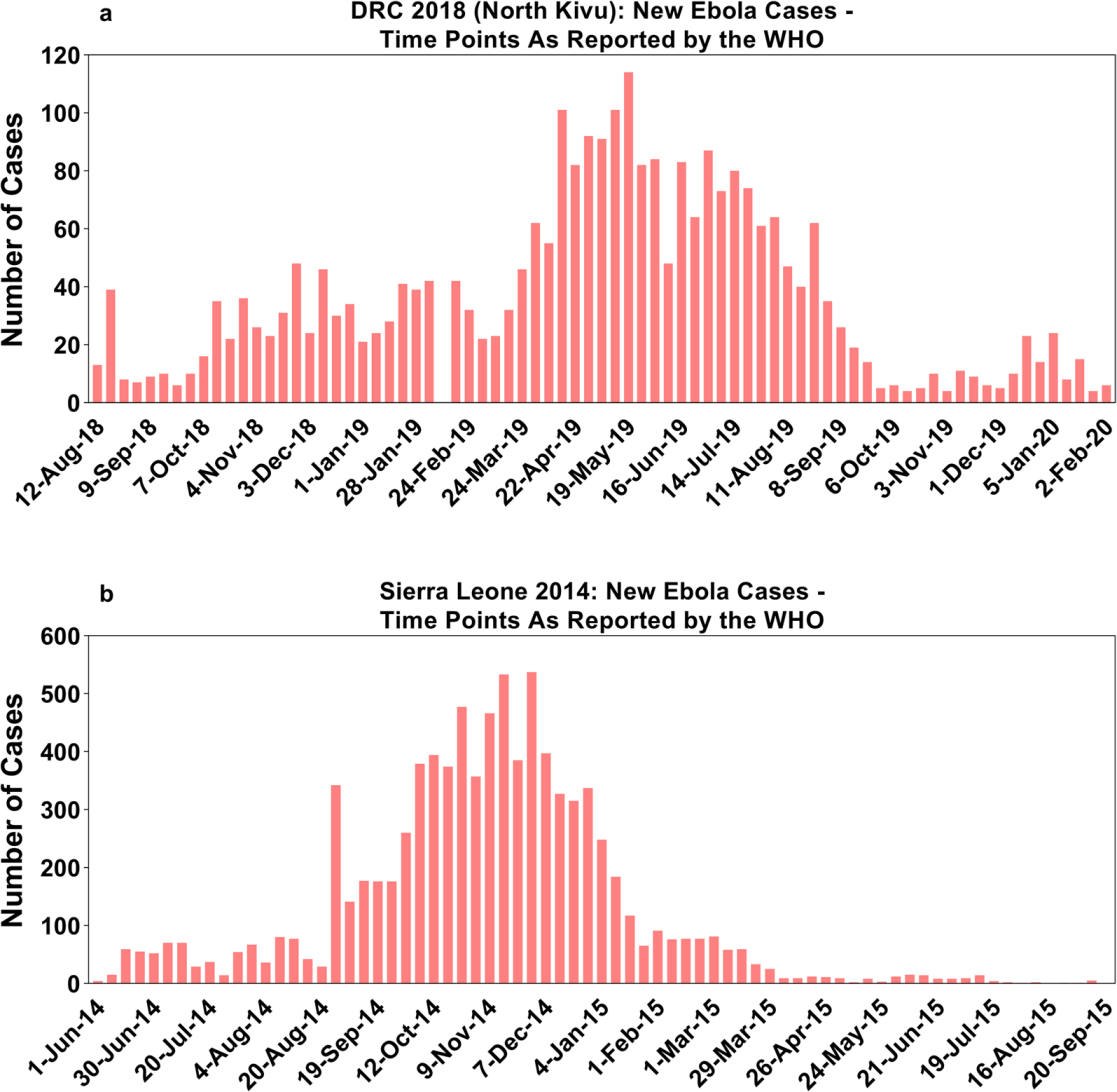
Comparison of weekly Ebola cases for epidemic of DRC (North Kivu) 2018 vs Sierra Leone 2014 as reported by the WHO

There were some key differences in outbreak characteristics between the 2014 SL epidemic and the 2018 DRC (North Kivu) epidemic. While the number of cases per million population was more in the 2014 SL epidemic as compared to the 2018 DRC (North Kivu) epidemic (1243 vs. 336), the epidemic duration was longer in the latter (> 99% of all cases were reported in the first 518 days vs 370 days in SL) (Table 6 and Fig. 8). The lower case penetration in the population in the DRC epidemic could be attributed to greater and faster use of ring vaccination leading to reduction in the effective reproduction number, although it suffered from multiple waves of outbreak in various geographical pockets.

**Table 6:** Model based on DRC 2018 (North Kivu) vs Sierra Leone 2014: comparison of underlying data

Parameter	Sierra Leone 2014	DRC 2018 (North Kivu)
Population	Total 7,017,144; HCW 1153	Total 8,300,000; HCW 5230
Overall cases	8704 cases over 480 days as reported by WHO; we took a time horizon of 587 days as there were no cases/deaths reported after the first 480 days	2791 confirmed cases (February 4, 2020) over 546 days in North Kivu as reported by WHO
Overall deaths	3589 deaths as reported by WHO	1875 deaths as reported by WHO
HCW cases	296 cases as reported by Fang 2016	145 cases; derived for North Kivu based on HCW cases reported for DRC as a whole by the WHO
Number of periods for model fitting (calibration)	Model horizon was divided into three periods for fitting: 0–95, 95–186, and 186 to 587	Model horizon was divided into five periods for fitting: 0–120, 120–217, 217–273, 273–385 and 385–546
Duration of epidemic	> 99% cases were reported in 370 days	> 99% cases were reported in the first 518 days

DRC: Democratic Republic of the Congo; HCW: healthcare workers; WHO: world health organization

It was interesting to note that the percentage of cumulative EVD cases averted, as compared with the “no vaccination” scenario, was consistently higher in the DRC (North Kivu)-based model compared with the SL-based model (Table 7), despite the lower number of cases and deaths per million population. To understand possible reasons for the greater impact of prophylactic vaccination projected in the DRC (North Kivu)-based model, we carried out a sensitivity analysis in which the duration of the outbreak was reduced by 20% while retaining unchanged all other model parameters. The results suggest that the longer an outbreak is likely to last, stemming from a slower or less effective response to an epidemic, the greater the impact of vaccination (Table 8).

**Table 7:** Ebola cases averted vs ‘no vaccination’– Sierra Leone 2014 vs DRC 2018 (North Kivu) models

Vaccine efficacy	Model based on Sierra Leone 2014 data				Model based on DRC 2018 (North Kivu) data			
	30%		90%		30%		90%	
Non-HCW	HCW							
	H30	H90	H30	H90	H30	H90	H30	H90
G0	23%	56%	33%	71%	31%	66%	42%	79%
G5	56%		70%		64%		77%	
G10	74%		85%		80%		89%	

DRC: Democratic Republic of the Congo; HCW: Healthcare workers; H30: 30% HCW vaccinated; H90: 90% HCW vaccinated; G0: 0% Non-HCW vaccinated; G5: 5% Non-HCW vaccinated; G10: 10% Non-HCW vaccinated

**Table 8:** Sensitivity analysis—model with original data vs model with duration of epidemic reduced by 20%

	No reduction of days (original DRC)		
	No vaccination	H0G5 (Vaccine efficacy 30%)	H0G5 (Vaccine efficacy 90%)
Cumulative cases (IQR; 95%CI)	2782 (700–4026; 54–9637)	1383 (302–2005; 48–5149)	1028 (228–1467; 47–3910)
Proportion of cases averted vs no vaccination (IQR; 95%CI)		50% (48–55%; 13–58%)	63% (61–67%; 13–70%)

	20% reduction of days (DRC)		
	No vaccination	H0G5 (Vaccine efficacy 30%)	H0G5 (Vaccine efficacy 90%)
Cumulative cases (IQR; 95%CI)	1711 (498–2449; 56–5823)	957 (248–1352; 48–3368)	757 (202–1092; 44–2631)
Proportion of cases averted vs no vaccination (IQR; 95%CI)		44% (43–46%; 13–51%)	56% (54–59%; 21–61%)

DRC: Democratic Republic of the Congo; HCW: Healthcare workers; H0: 0% HCW vaccinated; G5: 5% Non-HCW vaccinated

### Study limitations

This study has a few limitations. First, common to all compartmental models, we assumed that the risk of infection is homogeneous within each population subgroup (HCW, GP) included in the model. Similarly, we have taken prophylactic vaccination to be carried out at the same rate within each population subgroup. Further, we have not differentiated between HCW and non-HCW in the journey from when they are exposed. In the real world, however, none of these assumptions may fully hold. In such instances, there could potentially be differences in the extent/duration of the disease spread estimated in the model compared to that in the real world. These differences are minimized by calibrating the model with historical data such that it smoothens any heterogeneity and makes the model correspond to disease dynamics at the population level. Given this, we expect the directionality of this assessment in terms of the impact of vaccination to hold, as seen in the results of the various sensitivity analyses presented previously [26]. Second, to achieve the levels of protection assumed in the various prophylactic vaccination evaluations, the actual number of vaccinations needed may be higher to account for (i) waning protection of a vaccine, potentially requiring use of booster vaccination, and (ii) turnover in HCW staff, resulting in new recruits not being immediately protected. Third, the impact of vaccinating HCW will be less pronounced in cases where there are fewer HCW infections, with a study by Robert et al. having documented that while infections among HCWs play an outsized role in some outbreaks, they do not play as much of a role in others [49]. This variability has been attributed to a combination of factors by the authors, who add that the role of HCW cannot be predicted in advance of an actual outbreak. Fourth, the following model inputs related to infected individuals have been considered at a composite level across all infected individuals: (i) the assumed rate of transmission of EVD from deceased individuals in our model was a composite of the rate of transmission from deaths in the community and in Ebola treatment units, (ii) access to healthcare (hospitalization) has been taken at the population level and not differentiated by socio-economic strata, (iii) case fatality rate is a composite of rate among hospitalized individuals and those that were not hospitalized. We expect that considering differential rates for these parameters would not have impacted the directionality of the findings. Fifth, estimates of HCW involved in disease management in our model were based on pre-2014 data for the epidemic in SL [11] and 2014 data for the epidemic in the DRC (North Kivu) [51], and did not include frontline workers or any HCW added to the system during either epidemic. Sixth, any results showing in the analysis with wide 95% credible intervals should be interpreted with caution. Finally, we have not evaluated the impact of outbreak response with ring vaccination explicitly, since (i) the mean-field compartmental model approach is not ideally suited to capture all the key dynamics of ring vaccination, (ii) ring vaccination is already established as a go-to strategy in the immediate aftermath of an outbreak, and (iii) the impact of ring vaccination implemented in the 2018 epidemic in DRC (North Kivu) has been captured during the fitting process and is thus assumed to be intrinsically accounted for within the base case.

## Conclusions

Our conclusions from this study evaluating the impact of prophylactic vaccination of varying proportions of HCW and the general population on the size of a potential Ebola outbreak are that: (i) prophylactic vaccination against EVD, whether in HCW alone or complemented by the general population, may have a meaningful impact on the size and mortality of an outbreak, even if the scale of vaccinations and vaccine efficacy are both modest; (ii) the primary target for vaccination should be HCW, given their frontline role and consequently greater susceptibility to infection with EVD; (iii) a prophylactic vaccine that, in addition to reducing the risk of infection, also reduces infectiousness and case fatality rate in infected cases, will be beneficial even when vaccine efficacy in reducing the risk of infection is not 100%.

## Supplementary Information

**Additional file 1. **The model structure and transitions for the enhanced model along with detailed derivation of R0.

## Data Availability

All data generated or analyzed during this study are included in this published article [and its supplementary information files].

## Abbreviations

CDC: Centers for Disease Control and Prevention
CFRs: Case fatality rates
DRC: Democratic Republic of the Congo
EBOV: Ebola virus
EMA: European Medicines Agency
EVD: Ebola virus disease
FDA: Food and Drug Administration
FLW: Frontline workers
GDP: Gross domestic product
GP: General population
HCW: Healthcare workers
HIV: Human immunodeficiency virus
MVA-BN: Modified Vaccinia Ankara-Bavarian Nordic
SAGE: Strategic Advisory Group of Experts
SEIR: Susceptible, exposed, infected, and removed
SL: Sierra Leone
VSV: Vesicular stomatitis virus
WHO: World Health Organization
