# Supplementary material for: Model-based evaluation of the impact of prophylactic vaccination applied to Ebola epidemics in Sierra Leone and Democratic Republic of Congo
Source: BMC Infect Dis. 2022 Oct 4;22:769. doi: 10.1186/s12879-022-07723-6 (PMC9529325; doi:10.1186/s12879-022-07723-6)
Supplement: Supplementary file 1 — Additional file 1. The model structure and transitions for the enhanced model along with detailed derivation of R0. [file 12879_2022_7723_MOESM1_ESM.docx]

**Supplementary material A1**

**Methods**

***Choice of model***

This original model, as explained previously (1), had twelve compartments categorizing the total population (Figure 1 in Main Manuscript). The susceptible population was divided into HCW (SHCW) and General Population (other than HCW) (SNHCW) to account for the higher risk of exposure to Ebola virus among HCW. This also helped in assessing the benefit of vaccination strategies where HCW and the general population are vaccinated at differential coverage.

This existing model was enhanced to factor in the added benefit of vaccination in terms of reduced risk of infectiousness and case fatality rate of EVD in case of vaccinated individuals who become infected despite vaccination. Each of the post-exposure compartments were replicated (Figure 3 in Main Manuscript): exposed (E), infectious but not hospitalized (I), infectious and hospitalized (H), recovered (R), dead but not buried (D), and dead and buried (B) – one set to accommodate individuals that were not previously vaccinated or vaccinated but exposed prior to onset of efficacy (marked with suffix 1) and the other set to account for individuals who were previously vaccinated but exposed after onset of efficacy (marked with suffix 2).

The population (N) of the modeled geography at any given time is the aggregate of populations of all compartments

N = S_NCHW_ + S_HCW_ + V1 + V2 + V3 + V4 + E_1_ + I_1_ + H_1_ + D_1_ + R_1_+ B_1_+ E_2_ + I_2_ + H_2_ + D_2_ + R_2_+ B_2_

Where, S_NHCW_ = Susceptible population, general population other than healthcare workers (NHCW)

S_HCW_ = Susceptible population, healthcare workers (HCW)

V1 = Vaccinated but not yet protected HCW (prior to onset of efficacy)

V2 = Vaccinated and protected HCW (after onset of efficacy)

V3 = Vaccinated but not yet protected NHCW (prior to onset of efficacy)

V4 = Vaccinated and protected NHCW (after onset of efficacy)

E_1_ = Exposed with Ebola virus, non-vaccinated or non-protected (vaccinated but prior to onset of efficacy) individuals

I_1_ = Infectious with Ebola virus, non-vaccinated or non-protected individuals

H_1_ = Infectious and hospitalized, non-vaccinated or non-protected individuals

D_1_ = Infectious and dead but not buried, non-vaccinated or non-protected individuals

R_1_ = Recovered from Ebola, non-vaccinated or non-protected individuals

B_1_ = Dead and buried, non-vaccinated or non-protected individuals

E_2_ = Exposed with Ebola virus, vaccinated individuals post onset of efficacy

I_2_ = Infectious with Ebola virus, vaccinated individuals post onset of efficacy

H_2_ = Infectious and hospitalized, vaccinated individuals post onset of efficacy

D_2_ = Infectious and dead but not buried, vaccinated individuals post onset of efficacy

R_2_ = Recovered from Ebola, vaccinated individuals post onset of efficacy

B_2_ = Dead and buried, vaccinated individuals post onset of efficacy

At model initiation (t_0_), in the ‘no vaccination’ scenario, the entire population was initially considered susceptible, other than index infectious cases (compartment I_1_). The index infectious cases were considered to be 14 in the Sierra Leone (SL)-based model, which was in line with the 14 confirmed cases reported by the World Health Organization on May 29, 2014 for the epidemic in SL (2) and 16 in the DRC (North Kivu)-based model as reported by the World Health Organization on Aug 5, 2018 (3). The population in each individual compartment at any given point in time has been estimated by accounting for the inflows and outflows into each compartment since time t_0_. These flows were captured with the help of the expressions described in Supplementary Table 1 along with their pictorial depiction in Supplementary Figure 1 and the differential equations listed below. The description of vaccine related parameters used in these equations are provided in Supplementary Table 2, while the description of rest of the parameters have been explained above. Because of the relatively short time over which the epidemic has previously evolved, natural deaths and births were not considered in this model.

$$\frac{dS_{\mathrm{NHCW}}}{\mathrm{dt}}={-\xi_{2}S_{NHCW-}-\left( \frac{\left( (I_{1}+{ΩI}_{2})\beta_{I\to NHCW}+{(H}_{1}+{ΩH}_{2})\beta_{H\to NHCW}+(D_{1}+{ΩD}_{2})\beta_{D\to NHCW} \right)}{N} \right)S}_{\mathrm{NHCW}}$$

$$S_{\mathrm{NHCW}}\left( 0 \right)={S_{\mathrm{NHCW}}}_{0}\geq0$$

$$\frac{dS_{\mathrm{HCW}}}{\mathrm{dt}}=-\xi_{1}S_{\mathrm{HCW}}-\left( \frac{\left( (I_{1}+{ΩI}_{2})\beta_{I\to HCW}+{(H}_{1}+{ΩH}_{2})\beta_{H\to HCW}+(D_{1}+{ΩD}_{2})\beta_{D\to HCW} \right)}{N} \right)S_{\mathrm{HCW}}$$

$$S_{\mathrm{HCW}}\left( 0 \right)={S_{\mathrm{HCW}}}_{0}\geq0$$

$$\frac{dE_{1}}{\mathrm{dt}}=\frac{\left( (I_{1}+{ΩI}_{2})\beta_{I\to HCW}+{(H}_{1}+{ΩH}_{2})\beta_{H\to HCW}+(D_{1}+{ΩD}_{2})\beta_{D\to HCW} \right)}{N}(S_{HCW}+V_{1})+\frac{\left( (I_{1}+{ΩI}_{2})\beta_{I\to NHCW}+{(H}_{1}+{ΩH}_{2})\beta_{H\to NHCW}+(D_{1}+{ΩD}_{2})\beta_{D\to NHCW} \right)}{N}\left( S_{NHCW}+V_{3} \right)-\sigma E_{1} E_{1}\left( 0 \right)=E_{10}\geq0$$

$$\frac{dI_{1}}{\mathrm{dt}}=\sigma E_{1}-\alpha I_{1}-\delta_{1}\gamma I_{1}-\left( 1-\delta_{1} \right)\gamma I_{1} I_{1}\left( 0 \right)=I_{10}\geq0$$

$$\frac{dH_{1}}{\mathrm{dt}}=\alpha I_{1}{-(1-\delta}_{2})\gamma_{H}H_{1}-\delta_{2}\gamma_{H}H_{1} H_{1}\left( 0 \right)=H_{10}\geq0$$

$$\frac{dR_{1}}{\mathrm{dt}}=\left( 1-\delta_{1} \right)\gamma I_{1}+{(1-\delta}_{2})\gamma_{H}H_{1} R_{1}\left( 0 \right)=R_{10}\geq0$$

$$\frac{dD_{1}}{\mathrm{dt}}=\delta_{1}\gamma I_{1}+\delta_{2}\gamma_{H}H_{1}-\gamma_{D}D_{1} D_{1}\left( 0 \right)=D_{10}\geq0$$

$$\frac{dB_{1}}{\mathrm{dt}}=\gamma_{D}D_{1} B_{1}\left( 0 \right)=B_{10}\geq0$$

$$\frac{dE_{2}}{\mathrm{dt}}=\frac{\left( (I_{1}+{ΩI}_{2})\beta_{I\to HCW}+{(H}_{1}+{ΩH}_{2})\beta_{H\to HCW}+(D_{1}+{ΩD}_{2}{)\beta}_{D\to HCW} \right)}{N}\left( {\left( 1-\eta_{1} \right)V}_{2} \right)+\frac{\left( (I_{1}+{ΩI}_{2})\beta_{I\to NHCW}+{(H}_{1}+{ΩH}_{2})\beta_{H\to NHCW}+(D_{1}+{ΩD}_{2})\beta_{D\to NHCW} \right)}{N}({\left( 1-\eta_{2} \right)V}_{4})-\sigma E_{2} E_{2}\left( 0 \right)=E_{20}\geq0$$

$$\frac{dI_{2}}{\mathrm{dt}}=\sigma E_{2}-\alpha I_{2}-{\mu\delta}_{1}\gamma I_{2}-(1-{\mu\delta}_{1})\gamma I_{2} I_{2}\left( 0 \right)=I_{20}\geq0$$

$$\frac{dH_{2}}{\mathrm{dt}}=\alpha{I_{2}-(1-\mu\delta}_{2})\gamma_{H}H_{2}-{\mu\delta}_{2}\gamma_{H}H_{2} H_{2}\left( 0 \right)=H_{20}\geq0$$

$$\frac{dR_{2}}{\mathrm{dt}}=\left( 1-\mu\delta_{1} \right)\gamma I_{2}+{(1-\mu\delta}_{2})\gamma_{H}H_{2} R_{2}\left( 0 \right)=R_{20}\geq0$$

$$\frac{dD_{2}}{\mathrm{dt}}=\mu\delta_{1}\gamma I_{2}+\mu\delta_{2}\gamma_{H}H_{2}-\gamma_{D}D_{2} D_{2}\left( 0 \right)=D_{20}\geq0$$

$$\frac{dB_{2}}{\mathrm{dt}}=\gamma_{D}D_{2} B_{2}\left( 0 \right)=B_{20}\geq0$$

$$\frac{dV_{1}}{\mathrm{dt}}=\xi_{1}S_{\mathrm{HCW}}-\frac{\left( (I_{1}+{ΩI}_{2})\beta_{I\to HCW}+{(H}_{1}+{ΩH}_{2})\beta_{H\to HCW}+(D_{1}+{ΩD}_{2}{)\beta}_{D\to HCW} \right)V_{1}}{N}-\phi_{1}V_{1} V_{1}\left( 0 \right)={V_{1}}_{0}\geq0$$

$$\frac{dV_{2}}{\mathrm{dt}}=\phi_{1}V_{1}-\frac{\left( (I_{1}+{ΩI}_{2})\beta_{I\to HCW}+{(H}_{1}+{ΩH}_{2})\beta_{H\to HCW}+(D_{1}+{ΩD}_{2}{)\beta}_{D\to HCW} \right){\left( 1-\eta_{1} \right)V}_{2}}{N}$$

$$V_{2}\left( 0 \right)={V_{2}}_{0}\geq0$$

$$\frac{dV_{3}}{\mathrm{dt}}=\xi_{2}S_{G}-\frac{\left( (I_{1}+{ΩI}_{2})\beta_{I\to NHCW}+{(H}_{1}+{ΩH}_{2})\beta_{H\to NHCW}+(D_{1}+{ΩD}_{2})\beta_{D\to NHCW} \right)V_{3}}{N}-\phi_{2}V_{3} V_{3}\left( 0 \right)={V_{3}}_{0}\geq0$$

$$\frac{dV_{4}}{\mathrm{dt}}=\phi_{2}V_{3}-\frac{\left( (I_{1}+{ΩI}_{2})\beta_{I\to NHCW}+{(H}_{1}+{ΩH}_{2})\beta_{H\to NHCW}+(D_{1}+{ΩD}_{2})\beta_{D\to NHCW} \right){\left( 1-\eta_{2} \right)V}_{3}}{N}$$

$$V_{4}\left( 0 \right)={V_{4}}_{0}\geq0$$

**Supplementary Table 1. Transitions and expressions in the stochastic compartmental model.** For a pictorial depiction of the transitions, see Supplementary Figure 1**.**

| **#** | **Transition** | **Expressions** | **Transition due to:** |
| --- | --- | --- | --- |
| 1 | (S_HCW_, E_1_) →  (S_HCW_ − 1, E_1_ + 1) | $\left( \frac{\left( (I_{1}+{ΩI}_{2})\beta_{I\to HCW}+{(H}_{1}+{ΩH}_{2})\beta_{H\to HCW}+(D_{1}+{ΩD}_{2})\beta_{D\to HCW} \right)}{N} \right)S_{\mathrm{HCW}}$ | Infection of healthcare workers by infected (non-hospitalized), hospitalized, and dead but not buried individuals |
| 2 | (S_NHCW_, E_1_) → (S_NHCW_ − 1, E_1_ + 1) | $\left( \frac{\left( (I_{1}+{ΩI}_{2})\beta_{I\to NHCW}+{(H}_{1}+{ΩH}_{2})\beta_{H\to NHCW}+(D_{1}+{ΩD}_{2})\beta_{D\to NHCW} \right)}{N} \right)S_{\mathrm{NHCW}}$ | Infection of the general population (non-HCW) by infected (non-hospitalized), hospitalized, and dead but not buried individuals |
| 3 | (S_HCW_, V_1_) → (S_HCW_ − 1, V_1_ + 1) | $\xi_{1}S_{\mathrm{HCW}}$ | Vaccination of healthcare workers |
| 4 | (S_NHCW_, V_3_) → (S_NHCW_ − 1, V_3_ + 1) | $\xi_{2}S_{\mathrm{NHCW}}$ | Vaccination of the general population |
| 5 | (V_1_, V_2_) → (V_1_ -1, V_2_ +1) | $\phi_{1}V_{1}$ | Onset of efficacy of vaccine |
| 6 | (V_3_, V_4_) → (V_3_ -1, V_4_ +1) | $\phi_{2}V_{3}$ | Onset of efficacy of vaccine |
| 7 | (V_1_, E_1_) → (V_1_ -1, E_1_ +1) | $\frac{\left( (I_{1}+{ΩI}_{2})\beta_{I\to HCW}+{(H}_{1}+{ΩH}_{2})\beta_{H\to HCW}+(D_{1}+{ΩD}_{2}{)\beta}_{D\to HCW} \right)V_{1}}{N}$ | Infection of vaccinated healthcare workers before onset of efficacy by infected (non-hospitalized), hospitalized, and dead but not buried individuals |
| 8 | (V_3_, E_1_) → (V_3_ -1, E_1_ +1) | $\frac{\left( (I_{1}+{ΩI}_{2})\beta_{I\to NHCW}+{(H}_{1}+{ΩH}_{2})\beta_{H\to NHCW}+(D_{1}+{ΩD}_{2})\beta_{D\to NHCW} \right)V_{3}}{N}$ | Infection of vaccinated general population (non-HCW) before onset of efficacy by infected (non-hospitalized), hospitalized, and dead but not buried individuals |
| 9 | (E_1_, I_1_) → (E_1_−1, I_1_ + 1) | σE_1_ | Onset of infectiousness after completion of the latency period for non-vaccinated or non-protected individuals |
| 10 | (I_1_, H_1_) → (I_1_−1, H_1_ + 1) | αI_1_ | Hospitalization of infectious non-vaccinated or non-protected individuals |
| 11 | (I_1_, R_1_) → (I_1_−1, R_1_ + 1) | $\left( 1-\delta_{1} \right)\gamma I$_1_ | Self-recovery of infectious non-vaccinated or non-protected individuals from disease |
| 12 | (I_1_, D_1_) → (I_1_−1, D_1_ + 1) | $\delta_{1}\gamma I$_1_ | Death of infectious non-vaccinated or non-protected individuals before hospitalization |
| 13 | (H_1_, R_1_) → (H_1_−1, R_1_ + 1) | ${(1-\delta}_{2})\gamma_{H}H$_1_ | Recovery of infectious non-vaccinated or non-protected individuals from disease after hospitalization |
| 14 | (H_1_, D_1_) → (H_1_−1, D_1_ + 1) | $\delta_{2}\gamma_{H}H$_1_ | Death during hospital stay for non-vaccinated or non-protected individuals |
| 15 | (D_1_, B_1_) → (D_1_−1, B_1_ + 1) | $\gamma_{D}D$_1_ | Burial/isolation of dead individuals for non-vaccinated or non-protected individuals |
| 16 | (V_2_,E_2_) → (V_2_–1, E_2_ + 1) | $\frac{\left( (I_{1}+{ΩI}_{2})\beta_{I\to HCW}+{(H}_{1}+{ΩH}_{2})\beta_{H\to HCW}+(D_{1}+{ΩD}_{2}{)\beta}_{D\to HCW} \right){\left( 1-\eta_{1} \right)V}_{2}}{N}$ | Infection of vaccinated healthcare workers after onset of efficacy by infected (non-hospitalized), hospitalized, and dead but not buried individuals |
| 17 | (V_4_, E_2_) → (V_4_–1, E_2_ + 1) | $\frac{\left( (I_{1}+{ΩI}_{2})\beta_{I\to NHCW}+{(H}_{1}+{ΩH}_{2})\beta_{H\to NHCW}+(D_{1}+{ΩD}_{2})\beta_{D\to NHCW} \right){\left( 1-\eta_{2} \right)V}_{3}}{N}$ | Infection of vaccinated general population (non-healthcare workers) after onset of efficacy by infected (non-hospitalized), hospitalized, and dead but not buried individuals |
| 18 | (E_2_, I_2_) → (E_2_−1, I_2_ + 1) | σE_2_ | Onset of infectiousness after completion of the latency period for vaccinated individuals |
| 19 | (I_2_, H_2_) → (I_2_−1, H_2_ + 1) | αI_2_ | Hospitalization of infectious vaccinated individuals |
| 20 | (I_2_, R_2_) → (I_2_−1, R_2_ + 1) | $(1-{\mu\delta}_{1})\gamma I_{2}$ | Self-recovery of infectious vaccinated individuals from disease |
| 21 | (I_2_, D_2_) → (I_2_−1, D_2_ + 1) | ${\mu\delta}_{1}\gamma I$_2_ | Death of infectious vaccinated individuals before hospitalization |
| 22 | (H_2_, R_2_) → (H_2_−1, R_2_ + 1) | ${(1-\mu\delta}_{2})\gamma_{H}H$_2_ | Recovery of infectious vaccinated individuals from disease after hospitalization |
| 23 | (H_2_, D_2_) → (H_2_−1, D_2_ + 1) | $\mu\delta_{2}\gamma_{H}H$_2_ | Death during hospital stay for vaccinated individuals |
| 24 | (D_2_, B_2_) → (D_2_−1, B_2_ + 1) | $\gamma_{D}D$_2_ | Burial/isolation of dead vaccinated individuals |

**Supplementary Figure 1. Transitions in the enhanced stochastic compartmental model.**

**
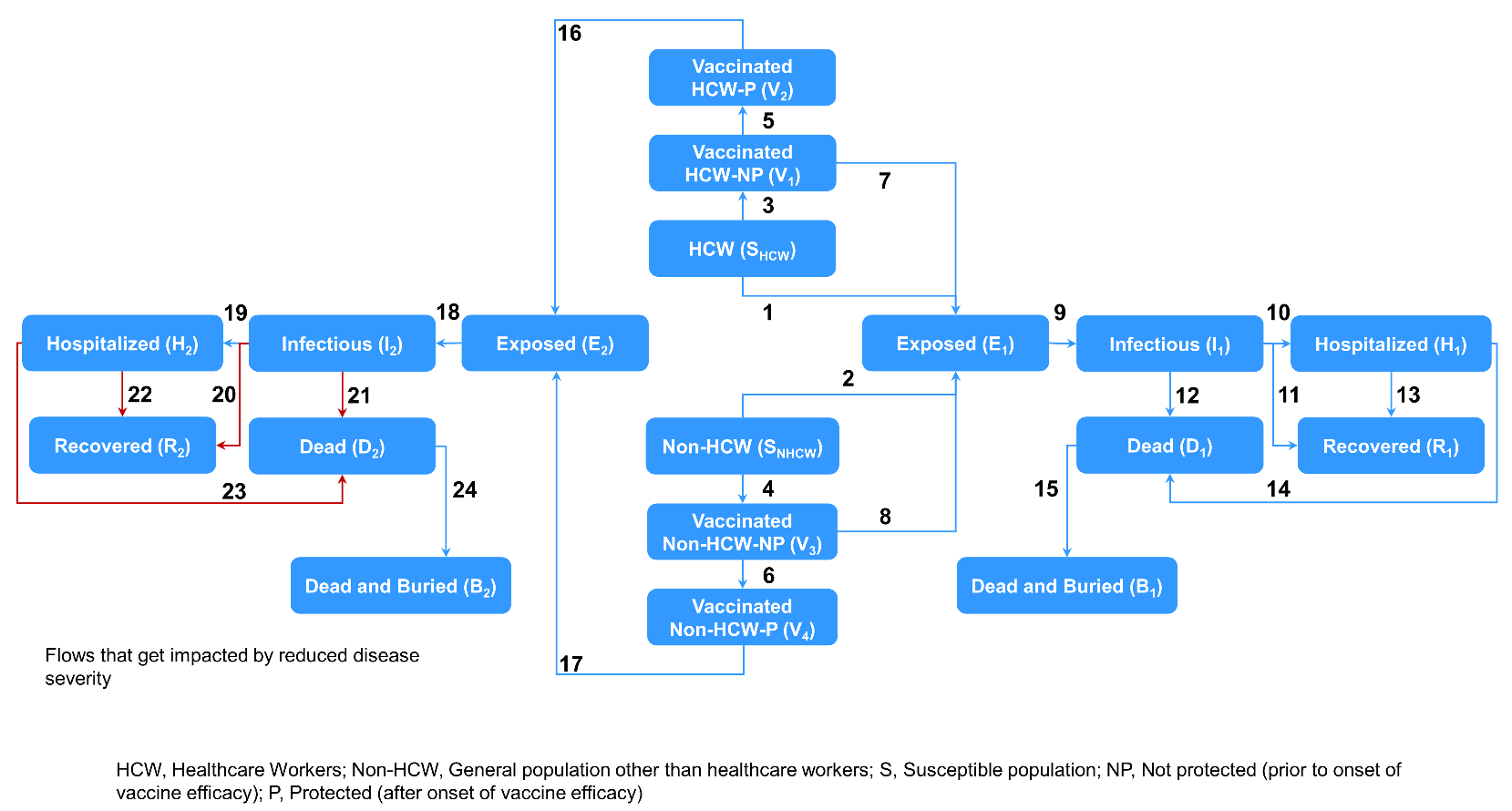
**

**Supplementary Table 2. Description of vaccine related parameters.**

| **Parameter** | **Description** |
| --- | --- |
| $Ω$ | % reduction in infectiousness for vaccinated protected individuals |
| $\mu$ | % reduction in case fatality rate of disease (case fatality rate in vaccinated individuals) |
| 1/ϕ | Time to onset of protection (in days) |
| ξ_1_ | Rate of vaccination in the HCW population (1/days) |
| ξ_2_ | Rate of vaccination in the general population, other than healthcare workers (1/days) |

In the enhanced model, there were no significant changes in the modeling approach compared to the original model; the only modification to the model was the addition of a separate set of compartments for vaccinated individuals. A deterministic mean-field compartmental model, based on the above equations but excluding terms relating to vaccination, was used to estimate the geographic/epidemic-specific parameters separately for pre- and post-intervention periods using a least square optimization technique. The model equations were also implemented using Gillespie’s direct method algorithm (4) to develop the stochastic mean-field compartmental model which was then used to simulate various vaccine strategies.

As part of the algorithm, two random numbers, R1 and R2, were generated from the uniform distribution in unit interval. A loop was then initiated involving the following steps: first, the probability of each of the n transitions occurring at the current time ($a_{i}$) was calculated; secondly, the time to the next event was determined as τ = (1/ a_0_)ln(1/R1) where a_0_ =$\sum_{i=1}^{n} a_{i}$, and the next event E_j_ was determined by the smallest integer j (where 1 < j < n) satisfying $\sum_{i=1}^{j} a_{i}> R_{2}a_{0}$. This next event was then executed by increasing the time by τ units and updating the population of the compartments impacted by the event. Finally, the loop was repeated until the end of the model time horizon. Results of various scenarios detailed in the main manuscript were presented as the cumulative cases and deaths, averaged across 5000 simulations.

**Basic Reproduction Number**

Given that there was no change in the parameters required for estimation of basic reproduction number, this remained the same as in the original analysis. The basic reproduction number (R_0_) was computed for the scenario without any intervention or vaccination (ξ1= ξ2=0), using a next generation matrix approach suggested by Diekmann and colleagues (5), represented by the equation:

$$R_{0}= \rho\left( {FV}^{-1} \right)$$

where ρ denotes the spectral radius, and the matrices F and V are transmission (spread of infection) and transition (movement between states), respectively. The F and V matrices are shown below, where the rows (from top to bottom) and columns (from left to right) represent exposed (E_1_), infected (I_1_), hospitalized (H_1_), and dead (D_1_) individuals, respectively:

Transmission Matrix F Transition Matrix V $\left[ \begin{matrix} . & E & I & H & D \\ E & 0 & {K_{1}\beta}_{I\to HCW}+\left( {1-K}_{1} \right)\beta_{I\to NHCW} & {{K_{1}\beta}_{H\to HCW}+}\left( {1-K}_{1} \right)\beta_{H\to NHCW} & \beta_{D} \\ I & 0 & 0 & 0 & 0 \\ H & 0 & 0 & 0 & 0 \\ D & 0 & 0 & 0 & 0 \end{matrix} \right] \left[ \begin{matrix} . & E & I & H & D \\ E & \sigma& 0 & 0 & 0 \\ I & -\sigma& \left( \alpha+\gamma\right) & 0 & 0 \\ H & 0 & -\alpha& \gamma_{H} & 0 \\ D & 0 & {-\delta}_{1}\gamma& {-\delta}_{2}\gamma_{H} & \gamma_{D} \end{matrix} \right]$

In transmission matrix F, HCW as a proportion of total population is denoted by K_1_. The model-fitted transmission rate *via* contact with infectious individuals was different for HCW (β_I🡪HCW_ in case of infectious individuals not hospitalized, and β_H🡪HCW_ in case of infectious individuals who are hospitalized) and the general population (β_I🡪NHCW_ in case of infectious individuals not hospitalized, and β_H🡪NHCW_ in case of infectious individuals who are hospitalized). However, the transmission rates *via* contact with individuals who were dead but not buried (β_D🡪HCW_= β_D🡪NHCW_= β_D_) were the same for both the HCW and general populations.

R_0_ was calculated as follows:

$$R_{0} = \left[ \frac{K_{1}\beta_{I\to HCW}+\left( 1-K_{1} \right)\beta_{I\to NHCW}}{\left( \alpha\right.+\left. \gamma\right)}+\alpha\left( \frac{K_{1}\beta_{H\to HCW}+\left( 1-K_{1} \right)\beta_{H\to NHCW}}{\gamma_{H}\left( \alpha\right.+\left. \gamma\right)} \right)+\frac{\beta_{D}\left( \delta_{1}\gamma+ \left. \delta_{2}\alpha\right) \right.}{\left( \alpha\right.+\left. \gamma\right)\gamma_{D}} \right]$$

$$R_{0}= R_{0I} +R_{0H}+R_{0D}$$

**References**

1. Potluri R, Kumar A, Maheshwari V, Smith C, Oriol Mathieu V, Luhn K, et al. Impact of prophylactic vaccination strategies on Ebola virus transmission: A modeling analysis. PLoS One. 2020;15(4):e0230406.

2. World health Organization (WHO). Ebola virus disease, West Africa – Update: 2014 - Guinea Geneva, Switzerland: World health Organization (WHO),; 2014 [updated May 28, 2014. Available from: <https://www.who.int/emergencies/disease-outbreak-news/item/2014_05_28_ebola-en>.

3. World Health Organization (WHO). Ebola Virus Disease - Democratic Republic of the Congo: External Situation Report 01. Brazzaville, Republic of Congo; 2018 August 7, 2018.

4. Gillespie DT. A general method for numerically simulating the stochastic time evolution of coupled chemical reactions. Journal of Computational Physics. 1976;22(4):403-34.

5. Diekmann O, Heesterbeek JA, Metz JA. On the definition and the computation of the basic reproduction ratio R_0_ in models for infectious diseases in heterogeneous populations. J Math Biol. 1990;28(4):365-82.
